# GIS model for geothermal advantageous target selection

**DOI:** 10.1038/s41598-023-32785-0

**Published:** 2023-04-13

**Authors:** Xuan Li, Changsheng Huang, Wei Chen, Yanan Li, Jihong Han, Xianguang Wang, Ximin Bai, Zhibin Yin, Xiaozhe Li, Pingping Hou, Jue Tong

**Affiliations:** 1 School of Environment Studies, China University of Geosciences, Wuhan, 430074 China; 2 Wuhan Center of China Geological Survey (Center South China Innovation Center for Geosciences), Wuhan, 430205 China; 3 Jiangxi Mineral Resources Guarantee Service Center, Nanchang, 330025 China; 4 Hydrogeological Brigade of Jiangxi Geological Bureau, Nanchang, 330095 China; 5 Institute of Geological Survey, China University of Geosciences, Wuhan, 430074 China; 6 Department of Jiangxi Province, The Fourth Geological Brigade of Jiangxi Geological BureauScience and Technology, Pingxiang, 337000 China

**Keywords:** Geochemistry, Geology, Geophysics, Hydrogeology

## Abstract

As the particularly popular green energy, geothermal resources are gradually favored by countries around the world, and the development model centered on geothermal dew point cannot meet the increasing geothermal demand. In this paper, a GIS model combining PCA and AHP is proposed, aiming to select the advantages of geothermal resources at the regional scale and analyze the main influencing indicators. Through the combination of the two methods, both data and empirical can be considered, then the geothermal advantage distribution on the area can be displayed through GIS software images. A multi-index evaluation system is established to qualitatively and quantitatively evaluate the mid-high temperature geothermal resources in Jiangxi Province, and carry out the evaluation of the dominant target areas and the analysis of geothermal impact indicators. The results show that it is divided into 7 geothermal resource potential areas and 38 geothermal advantage targets, and the determination of deep fault is the most critical index of geothermal distribution. This method is suitable for large-scale geothermal research, multi-index and multi-data model analysis and precise positioning of high-quality geothermal resource targets, which can meet the needs of geothermal research at the regional scale.

## Introduction

As a globally distributed green energy, geothermal resources have attracted attention from all countries, but only account for the smallest part of the total structure of renewable energy, which requires continued expansion of geothermal research depth and exploration. Geothermal research is not only limited to geothermal development, but also geothermal exploration, geothermal resource statistics, geothermal potential evaluation and so on^1–3^. Traditional geophysical and hydrogeological methods have an irreplaceable role, rich experience in traditional techniques can achieve good research results, such as using geophysical and hydrogeochemical methods^4^, judging by geological structure^5^, Using the geophysical and oil well information^6^, the traditional method of combining experience and data to realize the evaluation of geothermal potential in the corresponding area. But with the rise of space technology and the popularization of GIS since the twenty-first century, more and more scholars tend to build models to evaluate geothermal resources by means of GIS^7–9^.

Jiangxi Province is rich in geothermal resources along the southeastern coast of China. Bureau of Natural Resources of Jiangxi Province (government entity) has established geothermal data archives in the province, carried out geological exploration, geophysics, hydrology and infrared remote sensing^10^, and extremely rich basic geological data have been accumulated^11^. There are many influencing factors of geothermal^12–14^, and different combinations of index factors have also led researchers to innovate many evaluation models of different models^15^, and all of them have achieved corresponding results: the number and scope of geothermal distribution^16,17^ and terrestrial heat flow, magmatic rock distribution, faults Distribution, earthquake distribution, Bouguer gravity anomaly and magnetic anomaly distribution are closely related^18–21^.

Geothermal potential (Geothermal advantageous areas) is mainly a comprehensive assessment of the possibility of geothermal existence, geothermal temperature, and abundance of geothermal resources in a certain area. The purpose of this research is: (1) Evaluate the geothermal potential of Jiangxi Province using a combination of analytic hierarchy process (AHP) and principal component analysis (PCA). (2) Contrast and analyze the two methods, and analyze the important data of 12 indicators in three categories:(① Geothermal temperature: thermal storage temperature, including the Silicon enthalpy equation heat storage value, K/Mg cation temperature scale heat storage value; Geothermal water temperature. ② Geophysics: Moho feature, Curie feature, deep fault. ③ Basic geology: active fault, rifted basins, metamorphic rocks, magmatic rocks, earth heat flow value, and Radioactive heat generation of rocks). (3) Carry out the potential division of geothermal resources, and carry out geothermal advantageous target selection. (4) Count the high-value points in the typical geothermal resource target area, and compare and analyze the influence degree of each index in the geothermal optimal target area. Divide the entire Jiangxi into small units, classify, assign, and superimpose various impact indicators, and calculate the comprehensive value of each unit, so that the geothermal potential can be quantitatively evaluated.

### Study area

**Figure 1:**
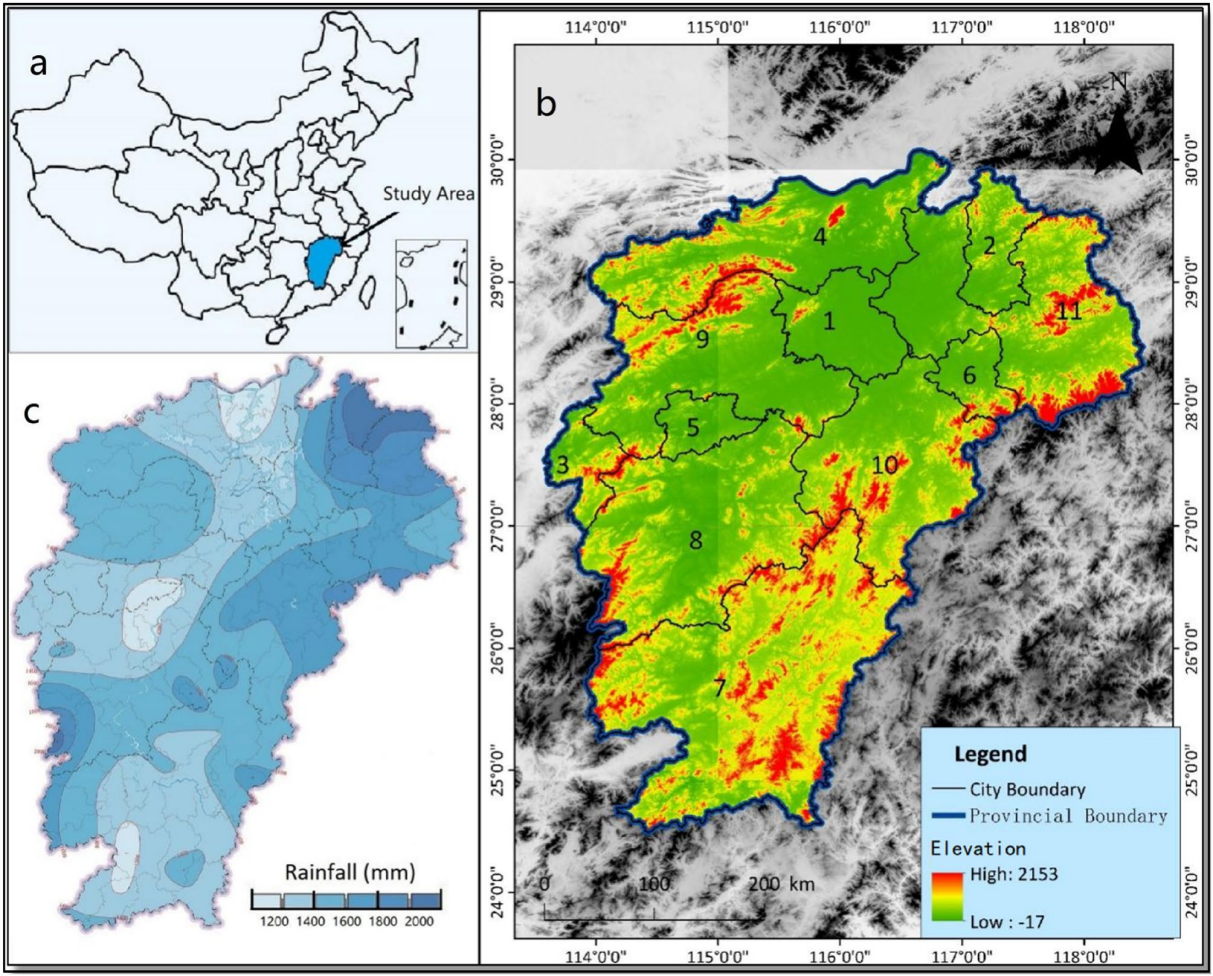
(**a**) Jiangxi Province is located in the southeastern coastal area of China. (**b**) The central areas of Jiangxi Province are two basins surrounded by mountains showing the distribution of major municipal cities; Red and green represent the elevation of the study area. (**c**) The annual average precipitation in Jiangxi Province^22^. 1—Nanchang; 2—Jingdezhen; 3—Pingxiang; 4—Jiujiang; 5—Xinyu; 6—Yingtan; 7—Ganzhou; 8—Jian; 9—Yichun; 10—Fuzhou; 11—Shangrao.

Jiangxi Province served as a case study area because data on its geothermal resources and associated regional-scale geoscience datasets are abundant and collectible. The study area is located in the central southeast of China, on the south bank of the middle and lower reaches of the Yangtze River, with a total area of 166,900 square kilometers. It is located between 24°29′ and 30°04′ north latitude and 113°34′–118°28′ east longitude. It borders Zhejiang and Fujian to the east, Guangdong to the south, Hunan to the west, and Hubei and Anhui to the north (Fig. 1).

The terrain is dominated by hills and mountains. The terrain of the province slopes to the north, with widespread basins and valleys. The northern part belongs to the Yangtze quasi-platform and the Jiangnan platform uplift, and the southern part belongs to the South China Fold System, forming a series of northeast-southwest trending structural belts, which belongs to an important geothermal outcropping zone. Fault depression basin with Poyang Lake as the center is formed in the northern area, geothermal exposure is evident at the edge of the basin. The climate is subtropical warm and humid monsoon climate, warm in winter and hot in summer. The average annual temperature is about 16.3–19.5 °C, and the annual precipitation is 1341–1943 mm.

There are 96 natural hot springs in the province, 32 geothermal waters discovered by drilling, and 127 geothermal hotspots in total. The highest hot spring temperature is 83 °C, the total amount of geothermal water resources is 113,317.6 m^3^/day, and the province's daily heat release is 1429.31 × 10^7^ kJ, equivalent to 488.98 t/day of standard coal. The heat resources in the province are divided into uplift mountain convection type and subsidence basin conduction type^23^. The former is distributed in the intermountain basins and piedmont areas of the folded and uplifted mountainous areas, and hot springs are often exposed on the surface; the latter are hidden in the subsidence basins, and hot springs are rare.

Jiangxi Province is located in the southeastern part of the Eurasian continental plate, adjacent to the Pacific plate, spanning the Yangtze and Huaxia ancient plates. Due to its proximity to the southeast coast and the Philippine Sea plate, the crustal uplift has been uneven in stages, and some faults have been active^24^. Since the Cretaceous, the crust of Jiangxi has become more brittle, and strong block faulting has occurred^25^, totaling more than 60. Magmatic activity occurred in all geological and historical periods in the study area, the volcanic rocks are most developed in the Yangtze and Yanshan periods^26^. Regional metamorphic rocks also exist that are also associated with geothermal activity: metamorphic core complexes, thermal domes, and blue schists^27^.

### Data compilation

We collected data from a variety of published sources, including the Wuhan Geological Survey Center and journal articles. This work has comprehensively collected geophysics, hydrochemistry, satellite remote sensing and other data from various government departments such as Jiangxi Provincial Department of Water Resources and Geological Bureau. All the figures are drawn with Arcgis 10.8 software.

On the basis of the previous work, we have traveled to Shicheng, Xiushui, Wugongshan, Suichuan, Sannan to carry out typical geothermal resource analysis and field monitoring experiments. These previous works include: depth data for Moho features, image; Curing feature depth data, image; Distribution data of Deep fault, image; Distribution data of Active fault, image; Distribution data of Rifted basins, image; Distribution data of Magmatic rocks and Metamorphic rocks, text and image; Regional numerical data of Earth heat flow value, image; Distribution data of radioactive heat generation of rocks, image. These are the data collected from this work.Geothermal thermal storage calculation data and geothermal water temperature data belong to the data collected in this work, and they are displayed in the image format after processing. The data comes from the Jiangxi Geological Exploration Fund Project^28^.

## Methods

The principal component analysis method can eliminate the correlation between evaluation indicators and reduce the workload of indicator selection. It reflects the proportion of the amount of information contained in the principal components in the original data to the total amount of information. In this way, determining the weight can better reflect the objectivity and rationality, and the calculation is more standardized, which is convenient for realization on the computer. The popularity of AHP is due to its simplicity, flexibility, ease of use, and interpretability^29,30^. AHP is a multi-objective, multi-criteria decision-making method capable of organizing, analyzing and solving complex decision-making problems^31^. Adjust the importance of each indicator through expert experience and group discussions, and prefer a semi-qualitative and semi-quantitative perspective. Comparing and demonstrating the potential of geothermal resources using two methods from different angles can not only ensure the objective and effective data results, but also obey the actual experience of experts and scholars. And through the comparison, the main factors affecting the distribution of geothermal resources can be seen more directly. The exploitation of mid-deep geothermal resources is of great risk and high cost, and blind drilling of wells will inevitably lead to losses. The comprehensive indicator method is a statistical analysis method that combines qualitative and quantitative analysis, and combines multiple factors for comprehensive evaluation^32^. The comprehensive index method refers to a method of calculating the comprehensive value of the individual index weighted average of each index on the basis of determining a reasonable index system for comprehensive evaluation.

### Principal component analysis

The principal component analysis method can screen out the main independent comprehensive factors from many variables, and at the same time retain the original main information, make them orthogonal to each other, which has advantages over the original variables^33^. On the premise of retaining the original amount of information as much as possible, PCA uses fewer dimensions to describe the original data information. The principal component analysis method first normalizes the original data, and establishes a correlation coefficient matrix and a principal component model. Then, the eigenvalues of the principal components of each indicator are obtained, and the weight of each indicator is obtained. In order to ensure that the selected indicators can scientifically and concisely reflect the advantageous areas of geothermal resources^34,35^. In this paper, the statistical software SPSS is used to carry out principal component analysis to calculate the weights of various indicators, and to determine the evaluation model of geothermal resource advantageous areas.

Principal component analysis was performed using IBM SPSS Statistics 24.0.0.0. (64). After importing the data table, use the "Analyze → Dimension Reduction → Factor "function in the main menu of the software to analyze.

Some software parameters need to be adjusted before the analysis begins. Import the 12 variables to be analyzed into the variable column, where parameters need to be set, including the "initial solution" option in the column of "Statistics", "coefficient", "KMO and Bartlett sphericity test", "reproduction" and "inverse image" option in the column of "correlation matrix".

Then follow the prompt box to carry out "Continue", "Extraction", "Rotation", "Scores" and "Options". Note the "Screen Graph" option in the "Display" bar, the Rotated solution and Loading plot(s) options in the Varimax option bar, the "regression" option in the "Method" TAB, Sorted by size and Suppress small coefficients options, enter "0.3".

When you finally view and analyze the output, you get a chart of the principal component analysis results for subsequent data analysis.

### Analytic Hierarchy Process

Analytic Hierarchy Process (AHP) refers to a decision-making method that decomposes decision-related indicators into levels such as goals, criteria, and plans, and then conducts qualitative and quantitative analysis on this basis. This method is a hierarchical weight decision analysis method proposed by American operations researcher Saaty (1980), a professor at the University of Pittsburgh, who applied network system theory and multi-objective comprehensive evaluation method^36^. AHP can reduce the subjective influence of human beings and better determine the weight of each evaluation indicator. AHP decomposes the problem into different components according to the nature of the problem and the overall goal to be achieved. And according to the interrelated influence and affiliation between the factors, the factors are aggregated and combined at different levels to form a multi-level analysis structure model, so that the problem can be boiled down to the lowest level (schemes for decision-making, measures, etc.) relative to the highest level (total target) relative importance weight or relative priority order^37,38^. A large number of practical cases prove that AHP has strong practicability in solving complex multi-objective competitive decision-making. AHP usually consists of four steps.

Step 1: Build a hierarchy. Hierarchies reveal the interrelationships of the various components of a complex problem. The determination of indicators mainly relies on the knowledge and experience of decision makers and relevant experts.
Step 2: According to the judgment of decision makers or experts, the relative importance of indicators is determined by pairwise comparison, and the judgment matrix Eq. (1) is constructed. Each indicator is assigned a value according to its impact size.

A_ij_ represents the importance of A_i_ to A_j_. The judgment matrix has the following properties: A_ij_ > 0; A_ij_ = 1/A_ji_; when i = j, A_ij_ = 1. Its value is shown in Table 1.

1 $$ A = \left[ {\begin{array}{*{20}c} {A_{11} } & \cdots & {A_{i1} } \\ \vdots & \ddots & \vdots \\ {A_{1j} } & \cdots & {A_{ij} } \\ \end{array} } \right] $$

**Table 1:** Rules for evaluating the importance of indicators in the analytic hierarchy process (AHP) matrix.

Importance	Meaning
1	Indicates that the factors i and j are equally important compared to the two factors
3	Indicates that the factor i is slightly more important than j
5	Indicates that the factor i is significantly more important than j
7	Indicates that the factor i is more important than j compared to the two factors
9	Indicates that the factor i is extremely important than j compared to the two factors
2, 4, 6, 8	The median value of the judgment of the above adjacent factors
Reciprocal	When i is compared with j, then the reciprocal A_ij_ = 1/A_ji_ of the comparison of factor j and i

Step 3: Calculate the weights through the judgment matrix to reflect the relative importance of these interrelated indicators. The maximum eigenvalue and eigenvector of the judgment matrix are obtained, and the eigenvector is the indicator weight. In order to prevent the value of the indicators in the judgment matrix A from changing too much, the indicators in the matrix are normalized to obtain a new matrix Bij, and the calculation method is shown in Eq. (2).
  2 $$ B_{ij} = \frac{{A_{ij} }}{{\sum\limits_{i = 1}^{n} {A_{ij} } }} $$

Equations (3) are used to compute the mean of the row indicators of the normalized matrix after computing the eigenvector matching the largest eigenvalue by B_ij_.

3 $$ w_{i} = \frac{{\sum\limits_{j = 1}^{n} {B_{ij} } }}{n} $$

The calculation of the largest eigenvalue is shown in Eq. (4).

4 $$ \lambda_{{{\text{max}}}} = \frac{1}{n}\sum\limits_{i = 1}^{n} {\frac{{\left( {Aw} \right)_{i} }}{{w_{i} }}} $$

In the formula: w_i_ is the average value of the row indicators of the normalized matrix, A is the initial matrix, n is the order of the matrix, and λ_max_ is the maximum eigenvalue.

Step 4: The random consistency indicator RI is related to the order of the judgment matrix. In general, the greater the order of the matrix, the greater the possibility of random deviation from consistency. Considering that deviations from consistency may be due to random reasons. Therefore, when testing whether the judgment matrix has satisfactory consistency, it is necessary to compare the consistency indicator CI (Eq. 5) with the random consistency indicator RI, and obtain the test coefficient CR. The formula is shown in Eq. (6) below. If CR < 0.1, it is considered that the judgment matrix passes the consistency test, otherwise it does not have satisfactory consistency.
  5 $$ CI = \frac{{\lambda_{{{\text{max}}}} - n}}{n - 1} $$
  6 $$ CR = {{CI} \mathord{\left/ {\vphantom {{CI} {RI}}} \right. \kern-0pt} {RI}} $$

In the formula: CI is the indicator of matrix inconsistency, CR is the random consistency ratio, RI is the average random consistency indicator, and n is the order of the matrix.

### Evaluation model

The geothermal resource potential evaluation model is established by the comprehensive indicator method: First, multiply the indicator that has been graded and assigned in each evaluation unit by the corresponding weight value. Then, the sum of all the indicator scores in the evaluation unit is calculated, and the comprehensive score of all the evaluation units is obtained in turn.

7 $$ DR = \sum\limits_{i = 1}^{P} {W_{i} \times Z_{i} } $$

In the formula: DR is the evaluation indicator of geothermal resource potential; Z_i_ is the standardized score of the indicator, and W_i_ is the weight of the corresponding indicator.

The comprehensive indicator value of geothermal resource potential evaluation is divided into 5 parts by the natural discontinuity method: resource advantage area, resource advantage area, resource general area, resource disadvantage area and resource disadvantage area.

### Indicator selection

According to the categories of factors affecting geothermal resources, it can be divided into three categories and a total of twelve indicators:

① Geothermal temperature: thermal storage temperature, including the Silicon enthalpy equation heat storage value^39^, K/Mg cation temperature scale heat storage value; Geothermal water temperature. ② Geophysics: Moho feature, Curie feature, deep fault. ③ Basic geology: active fault, rifted basins, metamorphic rocks, magmatic rocks, earth heat flow value, and Radioactive heat generation of rocks.

The corresponding evaluation system is established mainly based on these three types of factors, focusing on quantitative and semi-qualitative evaluation of the thermal resource potential in the region, and the key to the accuracy of the evaluation results lies in the selection and determination of evaluation parameters. Therefore, based on the existing application examples of geothermal resource evaluation, relevant evaluation parameters are selected and specified:

1. Thermal storage temperature: Thermal storage temperature is an important parameter to measure the ground temperature field^40^. The thermal reservoir temperature in the study area was calculated by the silicon enthalpy equation method and the K/Mg cation geothermal temperature scale method. The higher the thermal storage value, the more conducive to the exploitation and utilization of geothermal resources^41,42^.

Fournier envisages the method of quantitative chemical eothermometer, the mixing process of hot and cold water will inevitably lead to the initial enthalpy and SiO_2_ initial content of deep hot water falling to the final enthalpy and SiO_2_ content of spring water, so as to determine the initial enthalpy and SiO_2_ initial content of deep hot water through qualitative and quantitative calculations through the relationship between SiO_2_ and temperature. The silicon enthalpy equation method can use the mixed method to check the proportion of cold water, so as to obtain the accurate underground hot water temperature^43^. Principle: Under hypothetical conditions, the initial enthalpy of underground hot water and the mixing ratio of cold water can be estimated according to the local cold water temperature and the corresponding silica content, as well as the water temperature and its silica content. The high temperature of thermal storage calculated by the silicon enthalpy equation method generally reaches 200 °C. The highest temperature was Xianrenjing Hot Spring in Zhantian Township, Ningdu County, with a temperature of 269.94 °C; The second highest temperature was Hawthorne Hot Spring in Qinjiang Town, Shicheng County, with a temperature of 262.22 °C. The main exposed strata in the area are the Neoproterozoic Nanhua-Sinian strata, the Mesozoic Cretaceous strata and the Cenozoic Quaternary strata. The two hot springs are relatively close to each other, and both are located on the contact zone between the faulted basin and the north–north-east fault. The Anyuan-Yingtan deep fault and the Shicheng-Xunwu fault zone are the main controlling faults. The igneous rocks and thermal metamorphic rocks of multiple ages are widely distributed.

The geothermal water in Shicheng Hot Spring was formed on the eastern edge of the red-bed basin in the Late Cretaceous, at the intersection of two basin-controlling silicification fault zones, NNE and NWW. The temperature is 42°–50°, the water output is 1285 m^3^/day, and the heat storage temperature is calculated as 262.22 °C; The geotectonic location of Ningdu Hot Spring is located on the western edge of North Wuyi, and the north side of Ningyu Depression borders the north side of Taoshan-Yushan Uplift. Faults are relatively developed in the region, mainly including large-scale compression-shear faults in northeast, north–north-east and near-north–south directions. The highest temperature is 83°, the water output is 500–600 m^3^/day, and the heat storage temperature is calculated as 269.94 °C.

**Figure 2:**
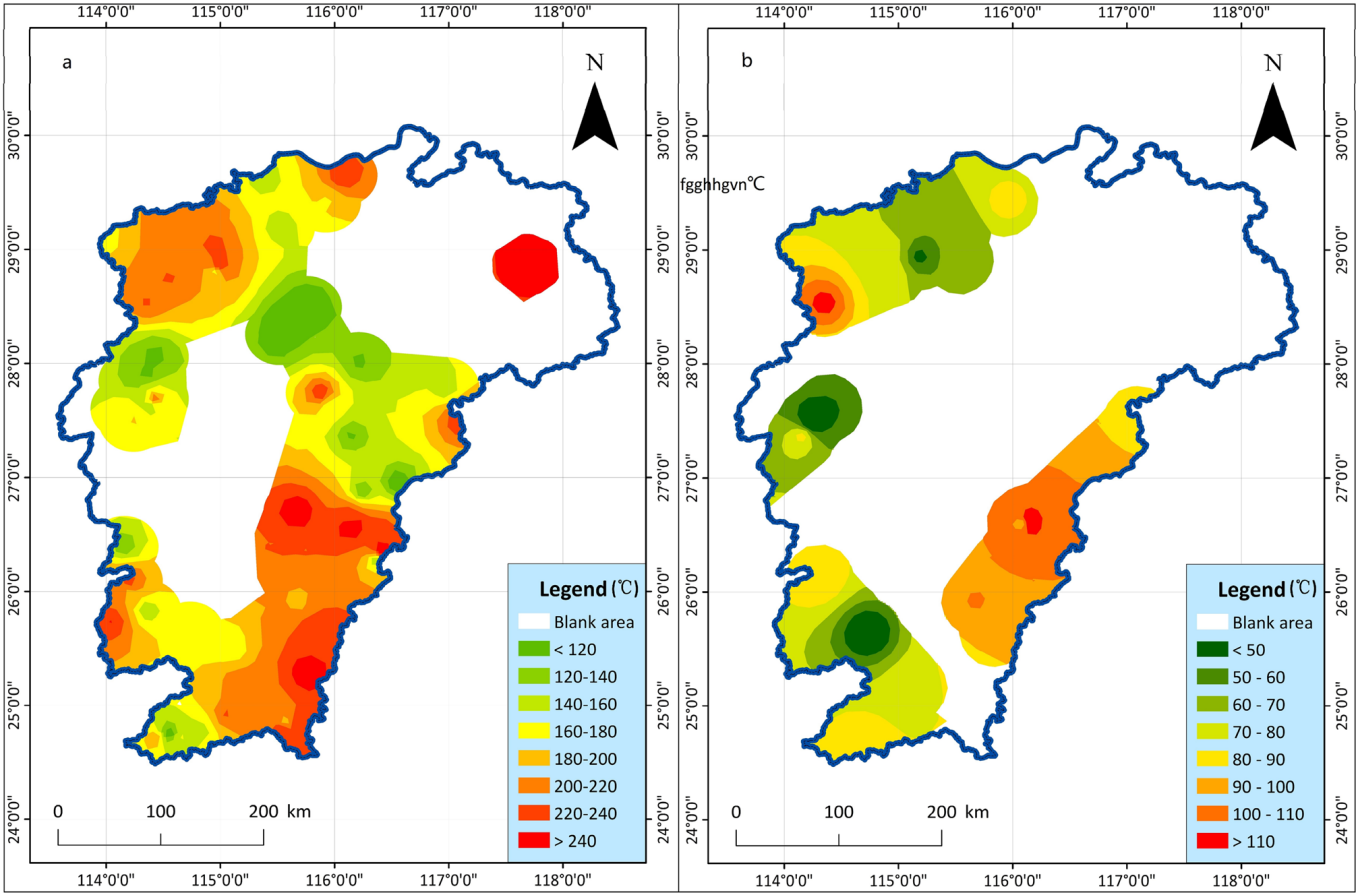
Displaying the thermal storage temperature distribution map is a comparison of two thermal storage calculation methods. (**a**) The thermal storage temperature distribution map of silicon enthalpy Eq. ^45^ heat storage value. (**b**) The thermal storage temperature distribution map of K/Mg cation temperature scale^46^ heat storage value.

The K/Mg geothermal temperature scale is based on the ion exchange reaction of potassium feldspar into muscovite and plagiochlorite, which is sensitive to changes in temperature^44^. When the hot water temperature decreases, the relative content of K^+^ and Mg^2+^ is adjusted rapidly. Therefore, the temperature estimated by this geothermal temperature scale may be the temperature of the shallower thermal storage. The areas with higher temperature are mainly concentrated in Jiuling and the vicinity of Shicheng-Guangchang, and it is more reasonable to verify with the temperature of artesian springs in this area. The thermal storage value of the silicon enthalpy equation and the K/Mg cation temperature scale thermal storage value are shown in Fig. 2.

2. Geothermal water temperature: The groundwater temperature is mainly monitored by artesian water and drilling sampling, which can clearly indicate the temperature of geothermal water in a certain place, and reflect the regional geothermal distribution to a certain extent^47^. The temperature of the geothermal water belongs to the group members independently collected data and then drawn. There are 118 exposed hot springs and exposed geothermal wells in the study area, and the measured geothermal temperature data obtained are compiled into geothermal temperature contours in Fig. 3. The highest temperature of the hot spring in the study area is 78 °C, located in Tanghu Town, Suichuan County; The highest temperature exposed by the geothermal well was 83 °C, located in Lantian Township, Ningdu County and Bailing Township, Xiushui County.

**Figure 3:**
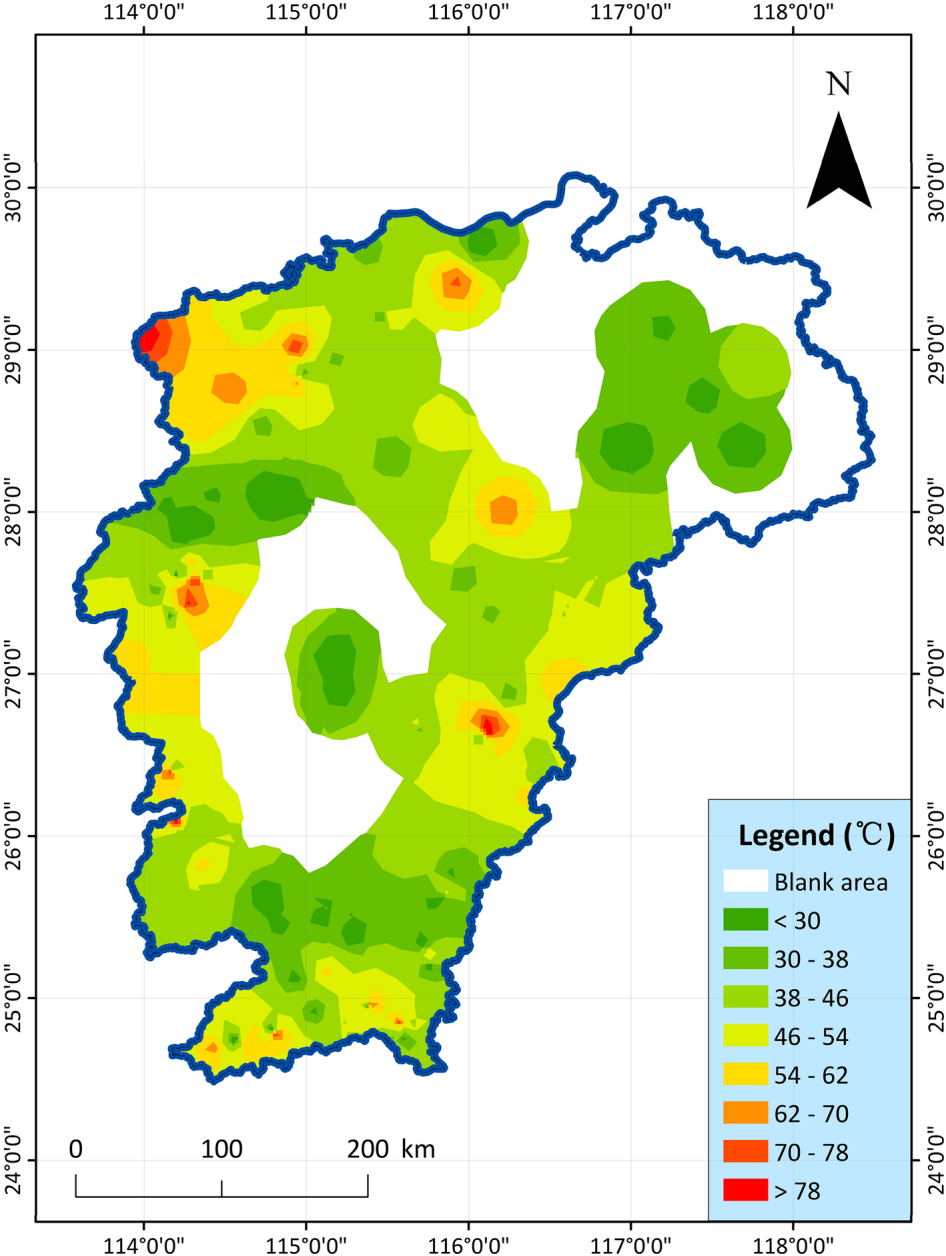
The temperature distribution map of naturally exposed hot springs or geothermal well water in Jiangxi Province^47^.
3. Moho feature: Moho surface is to use Bouguer gravity anomaly to study the undulation characteristics of Moho surface. The gravity field reflecting the Moho surface is extracted from the Bouguer gravity anomaly, which mainly reflects the undulation characteristics of the Moho surface^48^. The undulating characteristics of the Moho surface are similar to that of the Bouguer gravity anomaly extending upward for 30 km, showing the variation characteristics of shallow north and deep south, deep east and west, and shallow in the middle. Combined with the distribution of strata and magmatic rocks, the crust is thicker in the areas where magmatic rocks are developed, and the Moho surface is characterized by depression; In the sedimentary basin area, the crust is thin, and the Moho surface is characterized by uplift. The shallower the Moho surface, the richer the geothermal resources in general. The indicator data chart is shown in Fig. 4. For the geophysical and geological elements, we mainly use the aeromagnetic anomaly to study the Curie temperature isothermal surface, and the Bouguer gravity anomaly to study the Moho surface^49,50^.

**Figure 4:**
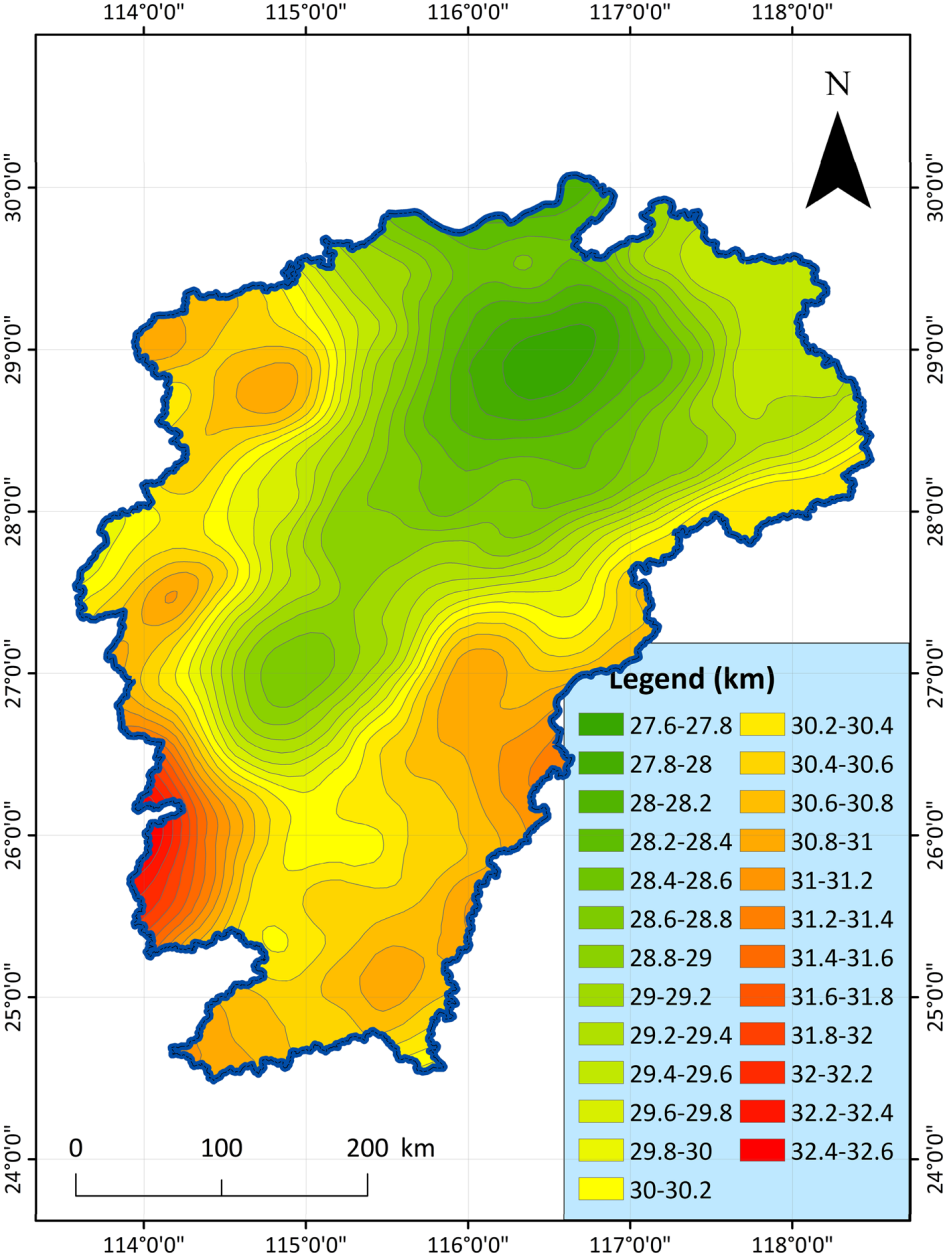
Distribution characteristic map of Moho depth in Jiangxi Province^51^.
4. Curing feature: Curing is an isothermal surface of about 578 °C. When the rock reaches the Curie temperature, it loses its ferromagnetism and turns into paramagnetism. Using aeromagnetic anomalies to study the Curie temperature isotherm is an effective method^52,53^. The aeromagnetization pole anomaly is extended upwards by 30 km to highlight the anomalous information of the deep field source and suppress or remove the local anomaly generated by the magnetic body above the Curie, which mainly reflects the fluctuation characteristics of the Curie. In addition, the areas with shallower burial depths generally have abundant geothermal resources. The index data are shown in Fig. 5.

**Figure 5:**
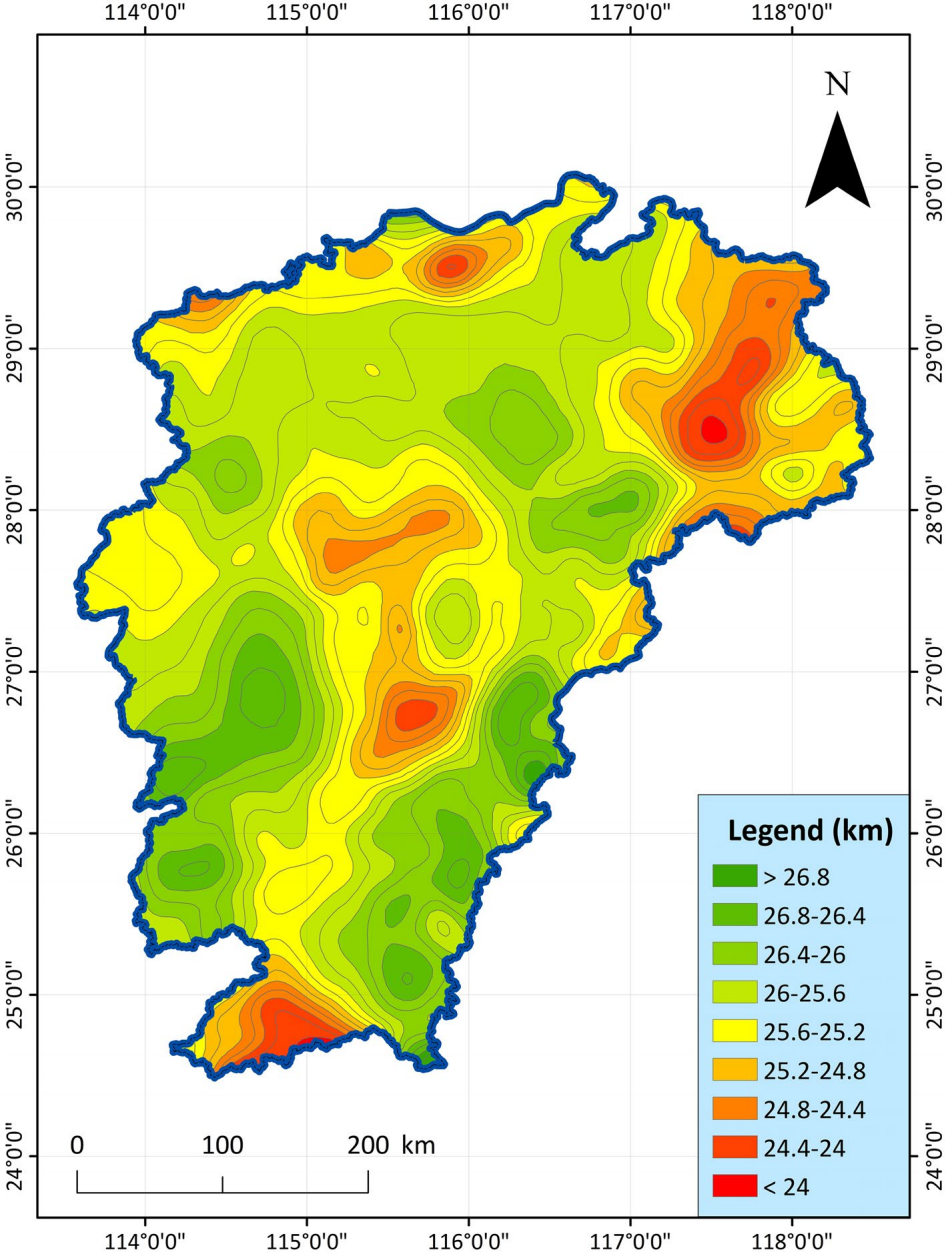
Distribution characteristic map of Curie depth in Jiangxi Province^51^.
5. Deep fault, Curie feature, deep structures: Use the remaining Bouguer gravity anomaly data to carry out notch analysis in the area, infer and explain the deep hidden or difficult to identify fault structures on the surface^54–56^. Under the regional background of the study area, the fault structures show the characteristics of NE-trending distribution. Directly using Bouguer gravity residual anomaly to carry out notch analysis, the obtained main linear structure also presents a NE distribution, and the secondary structure is a NW linear fault structure. The fault structure is the channel of heat transfer. The deep fault structure inferred and analyzed is drawn as Fig. 6. The geothermal probability is high in the distribution zone of these structures.

**Figure 6:**
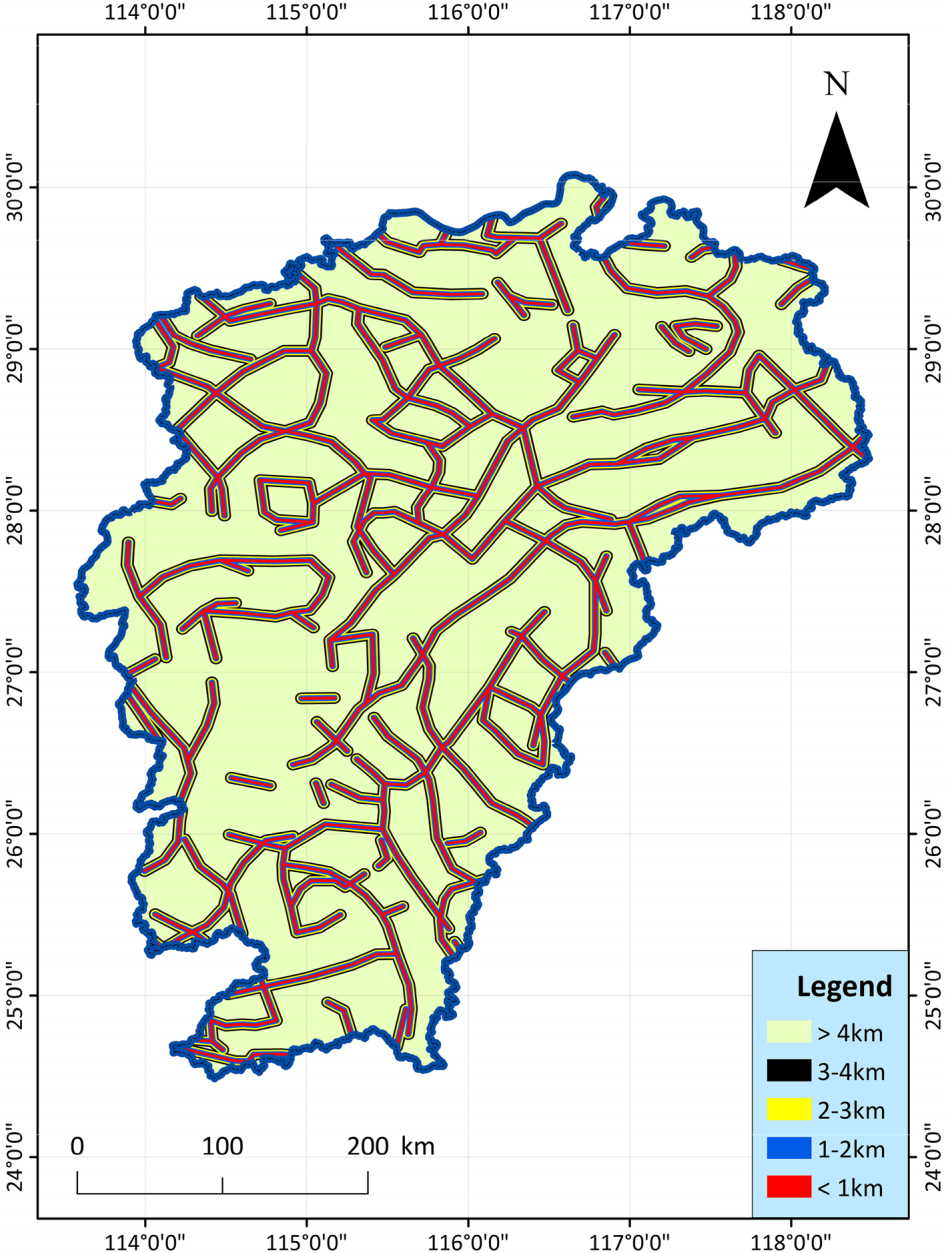
Spatial distribution map of deep faults and their distances from deep faults in Jiangxi Province^51^.
6. Active fault: In the recent geological period in Jiangxi, only depressions or wide and gentle folds occurred in the strata, with few faults and small scales. However, there are also some highly active faults cutting the edge of the basin, and the faults are often closely related to thermal conductivity and seismic activity. Faults are often channels for the transfer of geothermal and geothermal water, and geothermal resources are more likely to exist in these areas^57–59^. According to the data collected from geological surveys, aerial photographs and remote sensing ETM images, the main active fault belts in the study area in the recent period related to heat conduction and earthquake generation are as follows: NNE trending, NE trending fault tectonic belts, near-EW trending fault tectonic belts, and NW trending fault belts, the index data are shown in Fig. 7. The basic geological structure data such as active faults and rifted basins in the study area are mainly derived from the basic characteristics of the tectonic system in Jiangxi Province (the description of the tectonic system map)^60^.

**Figure 7:**
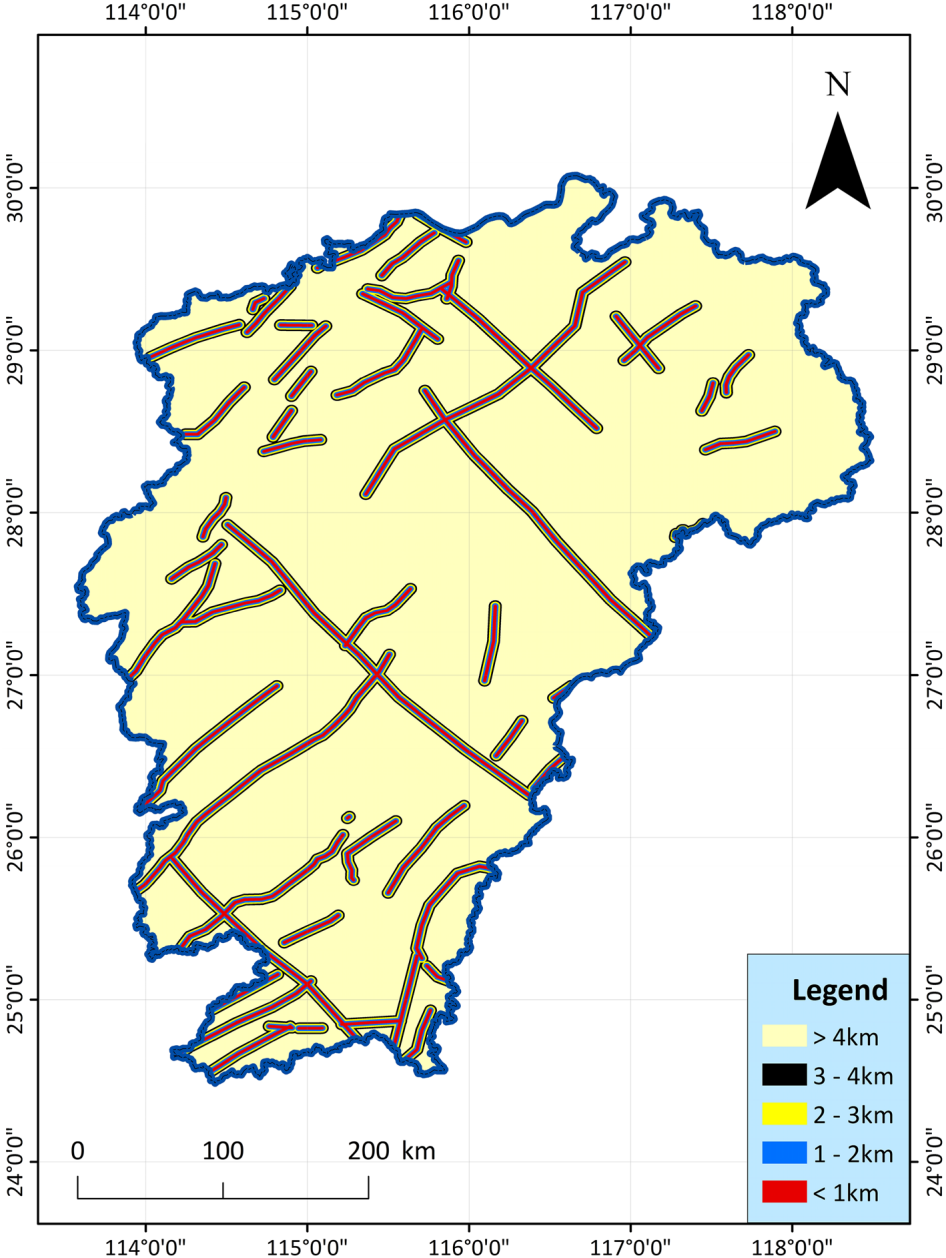
Spatial distribution map of active faults and their distances from active faults in Jiangxi Province^61^.
7. Rifted basins: On the tectonic background of the regional overall uplift, strong block faulting occurred in the study area, forming numerous continental faulted basins^62^, totaling more than 60. There are many small and medium-sized faults in the basin, and their distribution directions have the characteristics of NNE-trending belts, near-EW-trending lines, and NE-trending clusters. The fringes of faulted basins are sometimes overturned by later faults, and the formation of the basins has undergone the stress transformation process from extension to compression, and the regional geological structure is complex. In particular, there are many heat conduction channels in the basin boundary area, and geothermal resources are easier to find than non-faulted basin areas. The index data map is shown in Fig. 8.

**Figure 8:**
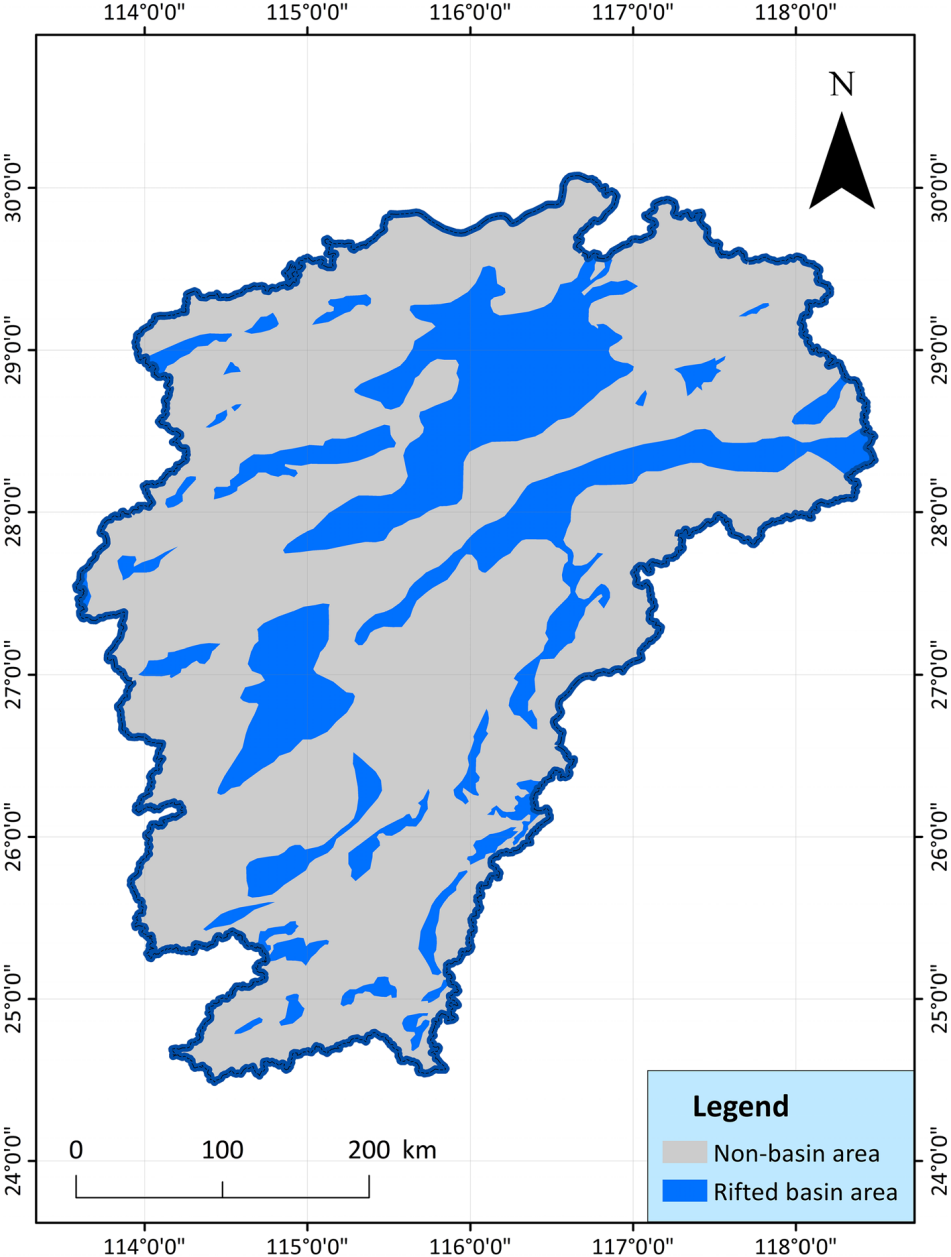
Spatial distribution map of Rifted basins in Jiangxi Province^61^.
8. Magmatic rocks: Magmatic rocks are developed in Jiangxi Province, and there are magmatic activities in various geohistorical periods. The volcanic rocks are most developed in the Yangtze and Yanshan periods. There are thousands of intrusive rock bodies in the whole province, the intrusive rocks are widely distributed, and there are many types of rocks, among which the acid rocks are the most developed. The shorter the time period for the formation of magmatic rocks, the higher the possibility of the existence of geothermal resources^28^. The distribution of magmatic rocks collected in the study area is shown in Fig. 9, which can help to infer the geothermal resource area. The data on the distribution characteristics of the relevant rock types (magmatic rocks, metamorphic rocks and Radiant rock mass) in the study area are mainly from the "China Regional Geology: Jiangxi Chronicle", and are compiled in combination with other documents^63^.

**Figure 9:**
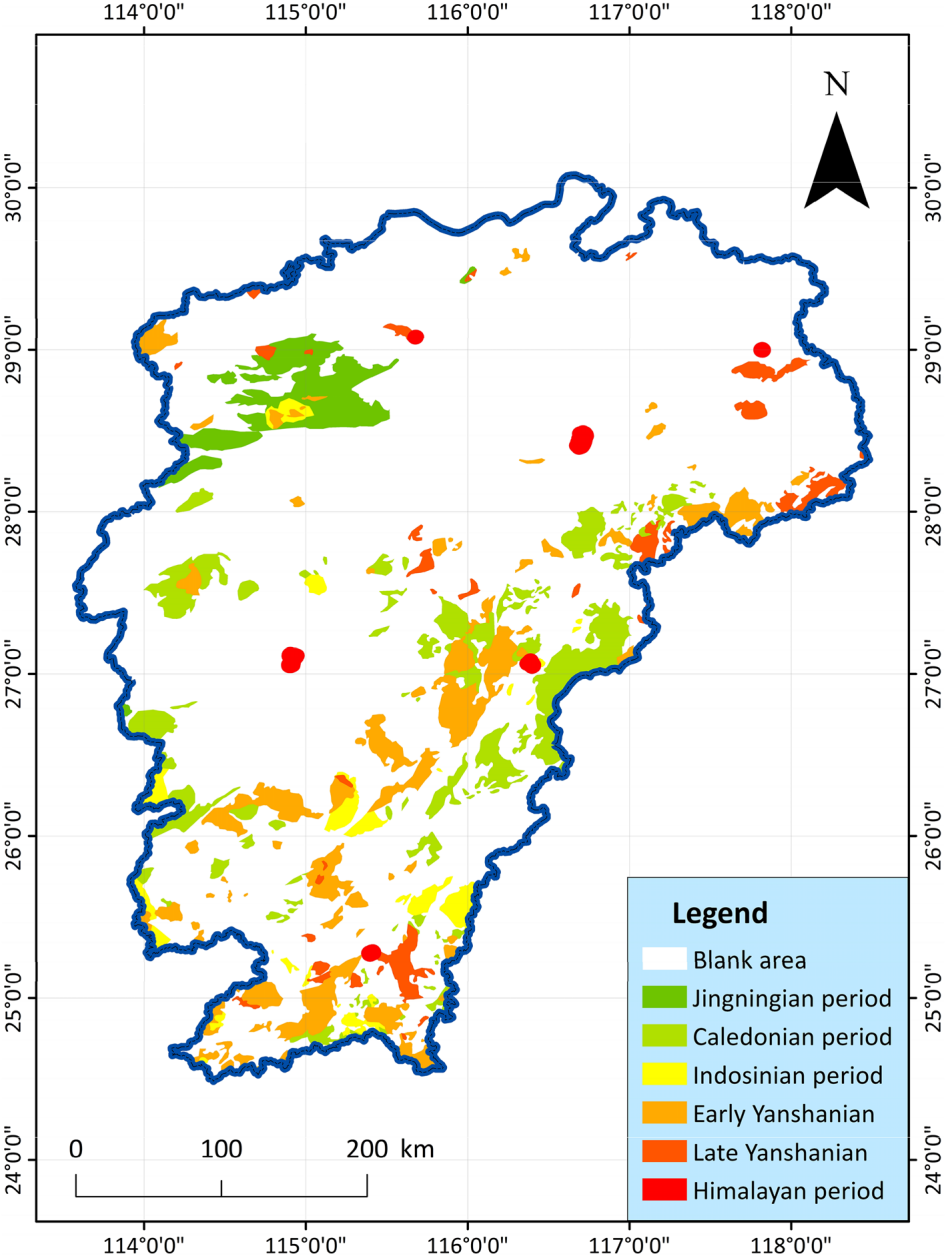
Spatial distribution map of Neoproterozoic-Cenozoic Era magmatic rocks in Jiangxi Province^64^.
9. Earth heat flow value: The heat transferred from the interior of the earth to the surface of the earth is called the geothermal flow. Geothermal heat flux is a comprehensive parameter, and it is the only physical quantity that can be directly measured on the surface of the earth's heat^65–67^. According to the contour map of China's terrestrial heat flow value drawn by the Geothermal Professional Committee of the China Energy Research Association, the terrestrial heat flow value in the study area is between 50 and 80 mW/m^2^ in most areas^68,69^. The higher the ground heat flow value, the higher the possibility of geothermal resources, the index data is shown in Fig. 10.

**Figure 10:**
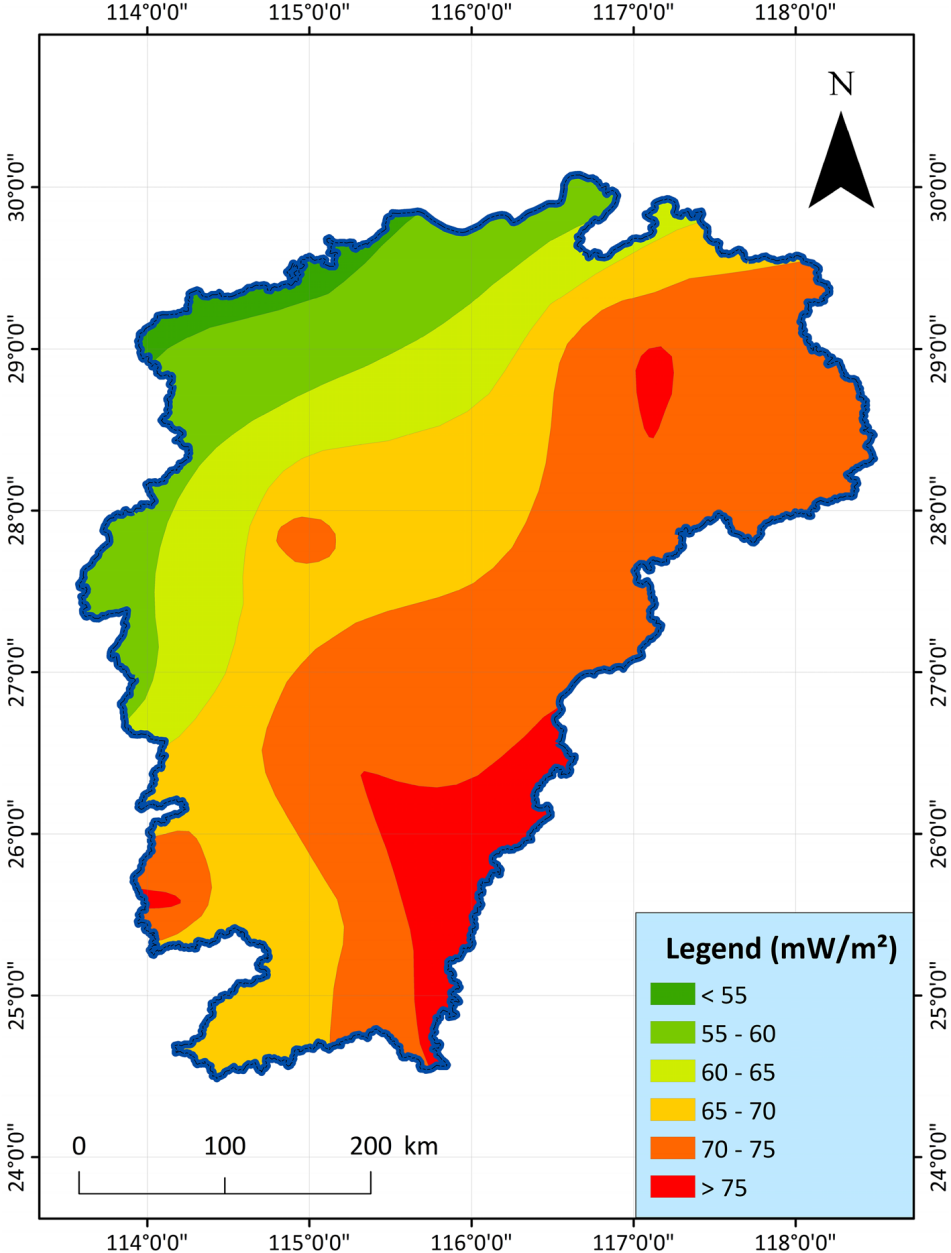
Distribution map of terrestrial heat flow value in Jiangxi Province^68^.
10. Radioactive heat generation of rocks: radioactive rock mass, mostly uranium ore, quartzite, etc., has the effect of radiant heating to surrounding rocks and groundwater. The heat generation rate of granite in the study area has the characteristics of "high in the south and low in the north, high in the east and low in the west", and these regions with radiative rock masses are more likely to be dominant geothermal resources. The index data is shown in Fig. 11.

**Figure 11:**
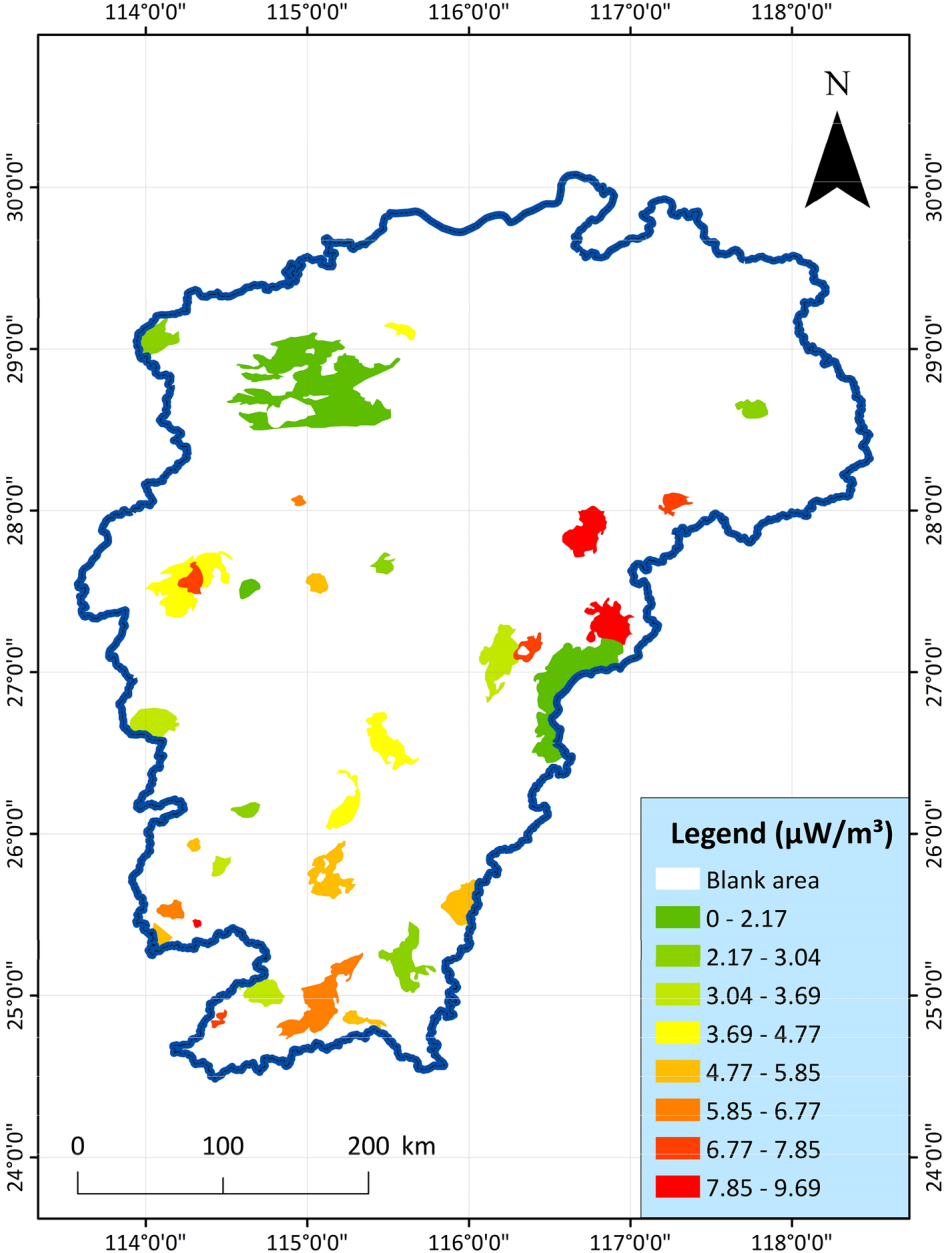
Distribution map of radioactive heat generation rate of acid magmatic rocks in Jiangxi Province^28^.
11. Metamorphic rocks: Under the influence of changes in environmental conditions, the mineral composition, chemical composition and structural structure of magmatic rocks or sedimentary rocks in the crust undergo metamorphism, thereby forming metamorphic rocks. According to the geological characteristics of the study area, the types of regional metamorphism are divided into regional low temperature dynamic metamorphism and regional dynamic thermal flow metamorphism. Metamorphic rocks formed by different types of action can reflect the geological activities in the region, which are mainly related to the activities of in-situ stress and the adjustment of heat flow in the crust and mantle^70^. The main types of metamorphic rocks in the study area are dynamic metamorphic rocks, metamorphic core complexes, thermal domes and blueschists, and their distribution characteristics are shown in Fig. 12.

**Figure 12:**
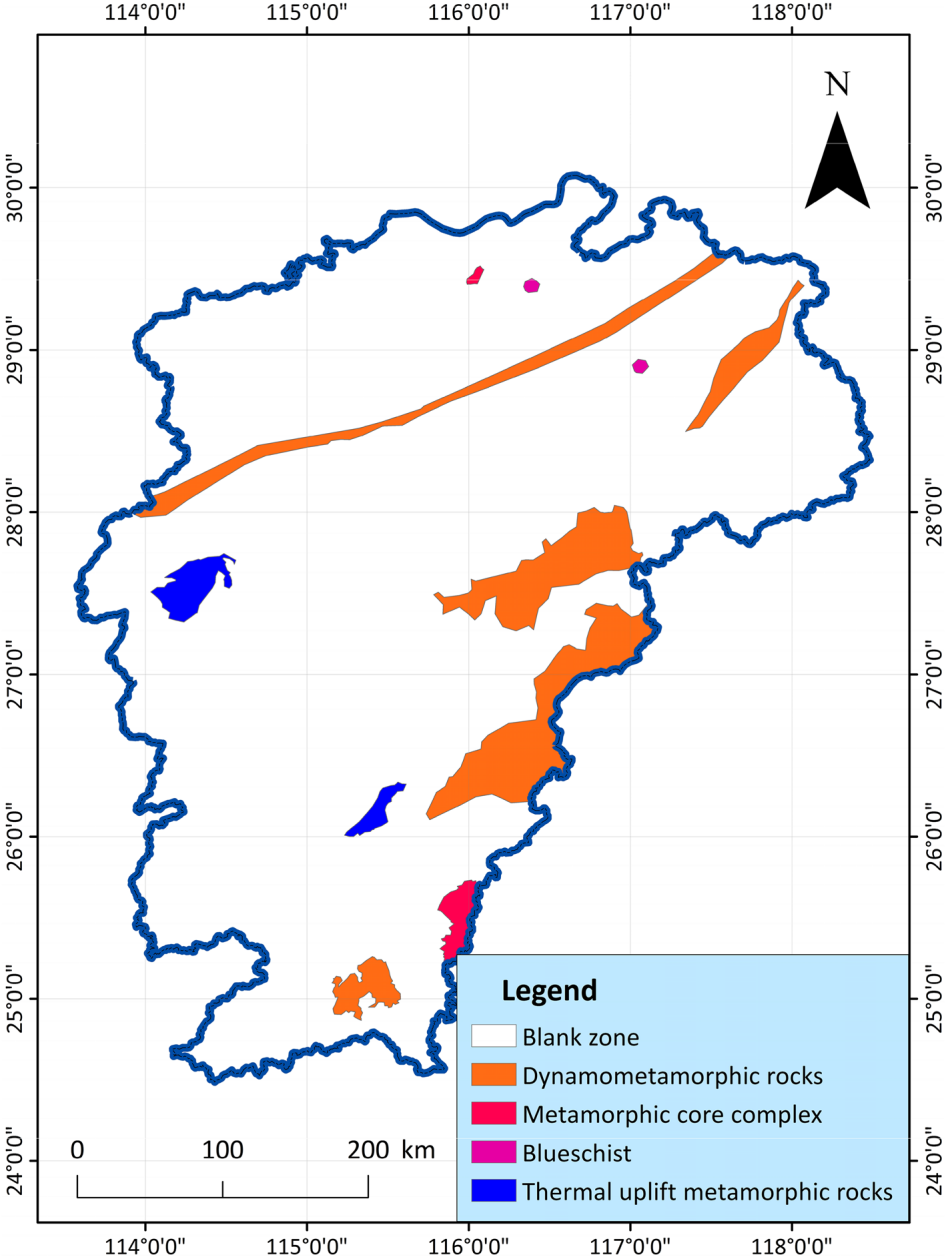
Distribution map of metamorphic rocks in Jiangxi Province^64^.

## Results

### Results of principal component analysis

Because there are many indicators for geothermal evaluation, and each attribute and value are not the same, it is necessary to standardize the original data of each indicator. In order to ensure the unity of the system of indicators, artificial classification is adopted here. And the evaluation indicator of geothermal resources should be assigned, which is divided into 9 levels as a whole, and the results of classification assignment are shown in Supplementary Table S1.

The principal component analysis was carried out after assigning the data of each evaluation indicator by the analysis software spss. The cumulative variance contribution rate of the first four principal components reached 54.261%, exceeding 50% (Supplementary Table S2 and Table 2). Four principal components were extracted according to the 50% cumulative variance contribution rate, and the variable with higher factor load was the indicator affecting geothermal resources.

**Table 2:** Component matrix of spss software principal component analysis (PCA) method.

Indicator	1	2	3	4
Moho feature	− 0.77	0.146	0.129	0.02
Silicon containing equation heat storage value	0.673	− 0.276	0.086	0.045
K/Mg cation temperature scale heat storage value	0.603	− 0.386	0.302	0.047
Magmatic rock	− 0.357	− 0.292	− 0.152	− 0.19
Rifted basin	0.311	0.775	− 0.16	− 0.007
Radioactive heat generation of rocks	0.261	0.532	− 0.16	− 0.273
Metamorphic rock	0.472	0.52	− 0.282	0.157
Curie feature	0.242	− 0.332	− 0.714	0.129
Earth heat flow value	0.543	− 0.021	0.574	− 0.101
Geothermal water temperature	− 0.295	0.418	0.429	0.217
Deep fault	0.001	0.032	0.061	0.856
Active fault	0.101	0.147	0.181	− 0.247

According to the corresponding variables and initial eigenvalues of each indicator in the principal component in the factor loading, the unit eigenvector is obtained according to formula  (4). Calculate the weight value of each environmental factor according to formula (3), and normalize it. It can be seen from Table 3 of the normalized weight results that the weight values of deep fault, metamorphic rocks, faulted basins, and terrestrial heat flow are relatively large. They are 0.1792, 0.2311, 0.2438, and 0.2518, respectively, which have a greater impact on the advantages of geothermal resources. Based on the weight value obtained by principal component analysis, the dominant factors affecting geothermal resources are deep fault, metamorphic rocks, faulted basins, and terrestrial heat flow.

**Table 3:** Indicator weights calculated by principal component analysis (PCA).

Indicator	Coefficients in the composite score model	Normalized weights
Moho feature	− 0.1264	− 0.1513
Silicon containing equation heat storage value	0.1248	0.1493
K/Mg cation temperature scale heat storage value	0.1242	0.1486
Magmatic rock	− 0.2009	− 0.2404
Rifted basin	0.2038	0.2438
Radioactive heat generation of rocks	0.1009	0.1207
Metamorphic rock	0.1931	0.2311
Curie feature	− 0.1176	− 0.1407
Earth heat flow value	0.2104	0.2518
Geothermal water temperature	0.1256	0.1503
Deep fault	0.1498	0.1792
Active fault	0.0481	0.0576

**Figure 13:**
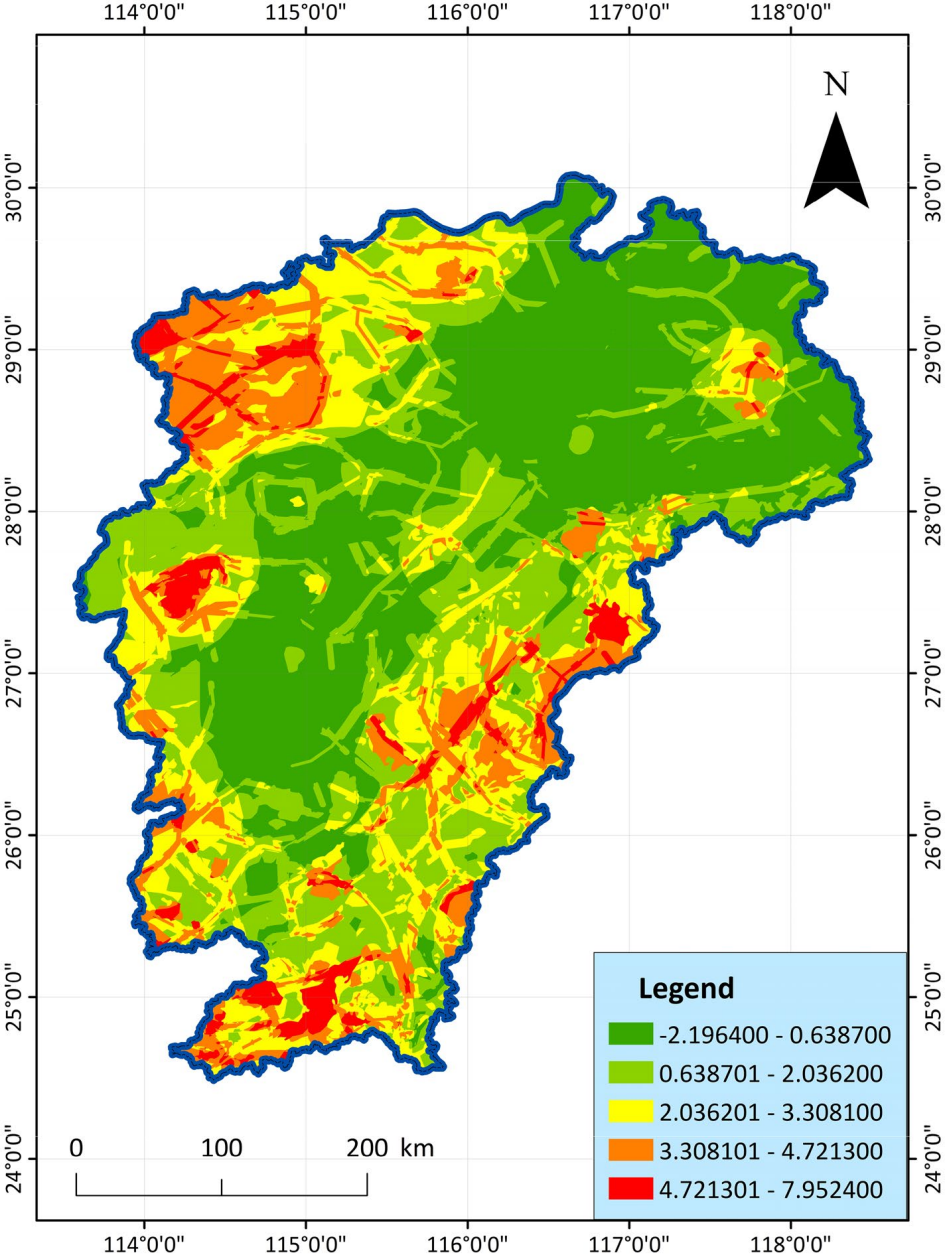
PCA geothermal resource potential evaluation diagram, showing the potential score for each cell.

Natural breakpoint method is a statistical method of grading and classification according to the numerical statistical distribution law, which can maximize the difference between classes, and is a data classification method that comes with the Arcgis software^71^. The weight value calculated by PCA is substituted into the comprehensive indicator method for superposition and calculation to obtain the geothermal resource potential evaluation indicator, and then divided into 5 grades by the natural breakpoint method: the dominant area (4.721301–7.952400), the better area (3.308101–4.721300), general area (2.036201–3.308100), poor area (0.638701–2.036200), inferior area (− 2.196400 to 0.638700), and finally the GIS software draws the composition area as shown in Fig. 13.

As shown in the figure, the dominant areas are mainly distributed in the western border, the southeastern border, and a small area in the north. The main ones are Jiuling, Xiushui, Wugong Mountain, Chongyi and Longnan in the west, Xunwu and Shicheng in the southeast, and a small part of Dexing in the north. These regions have high geothermal resources evaluation indicator and have high-quality geothermal resources development potential. In addition, some areas such as Jiuling and Shicheng have relatively high geothermal temperature and great development value, showing a state of overall distribution around Jiangxi. Green geothermal disadvantaged areas are mainly distributed in the central and northern regions of Jiangxi. The overall area rate is larger than that of the geothermal advantageous area, the geothermal development value is low, and the resources are not abundant.

### Results of Analytic Hierarchy Process

Based on discussion by all authors and multiple indicators judgment, the above 12 indicators are divided into three groups, and the indicators within the groups are of the same level. After preliminary discussion and analysis by all authors, a large number of indicators are initially classified, and then the weight of the indicators is accurately calculated through the AHP method to provide support for the subsequent comprehensive evaluation.

Group A (Silicon containing equation heat storage value, Geothermal water temperature, K/Mg cation temperature scale heat storage value), considering that the geothermal temperature is a direct performance factor, the geothermal enrichment in the region can be clearly pointed out, and its importance is divided into the highest level;

Group B (Curie feature, Deep fault, Active fault, Earth heat flow value, Radioactive heat generation of rocks) as basic geological elements, these elements affect the fundamental of geothermal formation and belong to the important basic elements of direct tectonic geothermal, and its importance is divided into sub-levels;

Group C (Rifted basin, Magmatic rock, Metamorphic rock, Moho feature) are indirect expression factors, not a direct expression of geothermal enrichment, but these elements have significant characteristics at the location of geothermal enrichment, which are indirect indicators of the lateral response to geothermal conditions, and their importance is divided into the lowest degree. Then, the judgment matrix of the three groups of ABC is constructed by formula (1), as shown in Table 4.

**Table 4:** Judgment matrix of AHP multi-level indicators.

Judgment matrix	Group A	Group B	Group C
Group A	1	2	3
Group B	1/2	1	2
Group C	1/3	1/2	1

Group A selects temperature-related index parameters, which can most directly indicate the possibility of geothermal existence and the richness of geothermal resources. Group B selects indicators that can indirectly reflect the geothermal situation, or vaguely indicate the geothermal distribution, and the accuracy of these indicators is relatively poor; Group C mainly characterizes the existence of traces of geothermal activity, which has a certain correlation with the existence of geothermal resources. AHP needs to first rank the importance of the indicators, and the judgment basis of the ranking is: the influence of the indicators on the occurrence of geothermal resources. Through the description and analysis of the above indicators, it can be judged that the importance of group A is the highest, group B is medium, and group C is the lowest.

Through formulas (2–4), the weight values of the three groups of indicators are calculated. And use formula (5, 6) to test to get the weight value and test coefficient as shown in Table 5.

**Table 5:** Weight values and test coefficients calculated by AHP.

Indicator group	Group A	Group B	Group C
Weights	0.5396	0.297	0.1634
Random consistency ratio = 0.0088 < 0.1, passed the consistency test, with satisfactory consistency			

Since the indicator grades in each group are judged the same, the analysis shows that the weights of each indicator are: the weights of indicators in group A were all 0.1799 (among which K/Mg cation temperature scale heat storage value was 0.1798); the weights of indicators in group B were all 0.0594; and the weights of indicators in group C were all 0.04085.

**Figure 14:**
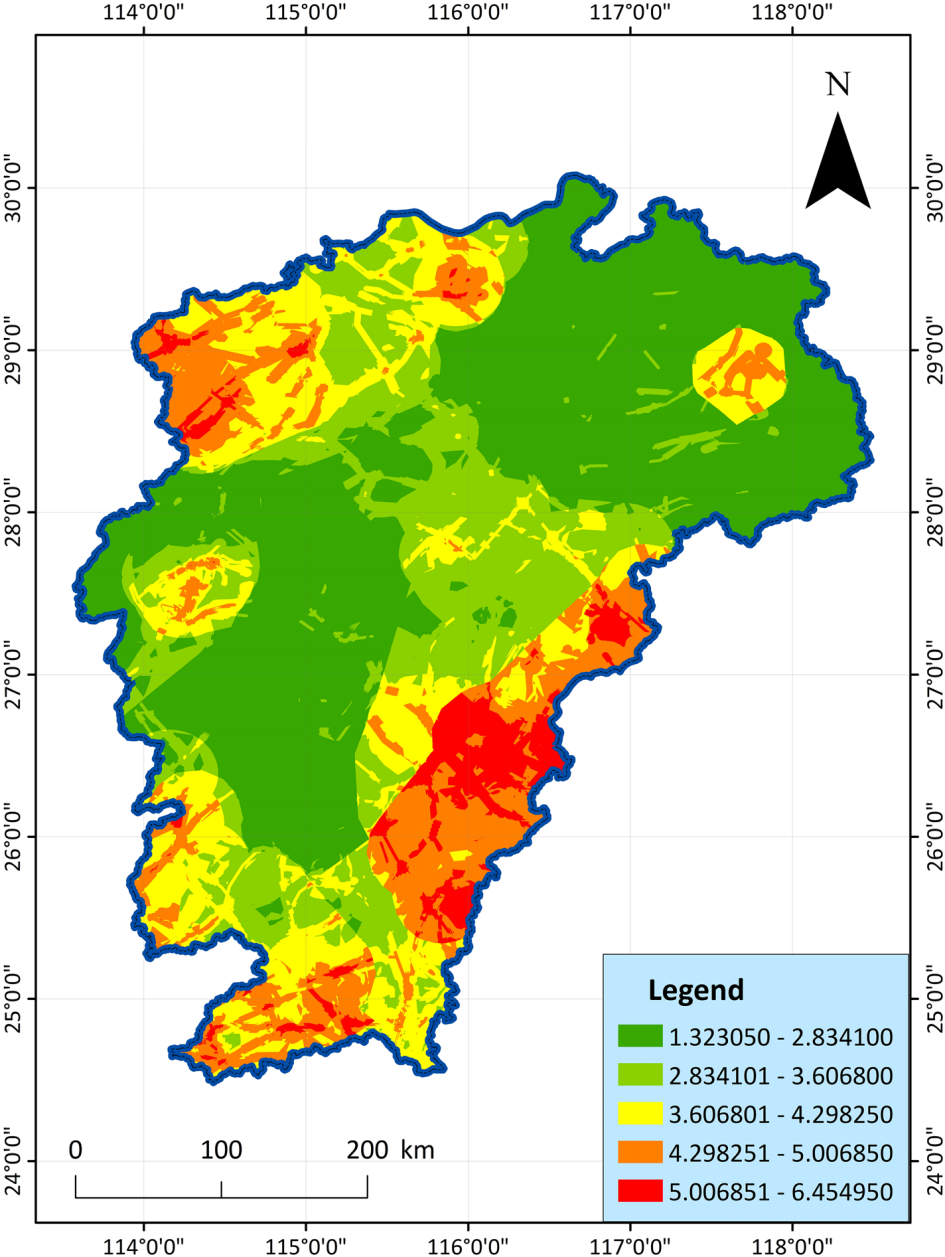
AHP geothermal resource potential evaluation diagram, showing the potential score for each cell.

The weight value calculated by AHP is substituted into the comprehensive indicator method for superposition and calculation, so as to obtain the evaluation indicator of geothermal resource potential, which is then divided into 5 grades by the natural breakpoint method: advantageous area (5.006851–6.454950), superior area (4.298251–5.006850), general area (3.606801–4.298250), poor area (2.834101–3.606800), inferior area (1.323050–2.834100), and finally the GIS software draws the composition area as shown in Fig. 14.

As shown in the figure, the dominant areas are mainly distributed in the border areas of Shicheng, Xunwu, Longnan and Chongyi in the southeastern border of Jiangxi. In the northern part of Jiangxi, Wugong Mountain, Jiuling, Xiushui and Dexing are also areas with rich geothermal resources. In addition, the above areas have been found to be rich in hot water springs and geothermal exploration wells, which are relatively concentrated in a certain area and have great development and utilization value, which is suitable for planning as a geothermal resource advantage area. Most areas in central and northern Jiangxi are disadvantaged areas with low geothermal evaluation indicator. In contrast, the area is large, but the geothermal distribution is small and resources are scarce. It is not recommended to carry out large-scale geothermal development in these areas.

### Method comparison

In the evaluation of geothermal resource potential, the determination of the weight is an important part. Once the empowerment is biased, it will directly lead to incorrect evaluation results. The spatial differences of geothermal resources make their characteristics, structures and performances different, and the main indicator factors that control, influence and reflect the advantages of geothermal resources are also different. First, use PCA to optimize the indicators, determine Moho feature, Silicon containing equation heat storage value, K/Mg cation temperature scale heat storage value, Magmatic rock, Rifted basin, Radioactive heat generation of rocks, Metamorphic rock, Curie feature, Earth heat flow value, Geothermal water temperature, Deep fault, Active fault, a total of 12 factors are the evaluation indicators of geothermal resource potential. Then, the indicator weight is normalized by the weighted average of the coefficients in the linear combination of the principal components. Then, each indicator is weighted and superimposed by the comprehensive indicator method, and the comprehensive indicator value of each evaluation unit is obtained.

Based on the characteristics of the PCA weighting method itself, the weight of each evaluation factor can be determined more accurately, which is in line with the measured values of each evaluation factor at the monitoring point, and can more accurately evaluate the geothermal resource advantageous area. Relatively speaking, AHP pays more attention to the opinions of experts, and can more effectively combine the actual situation and years of geothermal research experience. Between semi-quantitative and semi-qualitative, it can better reflect the actual impact of the indicator indicators, and can better reflect the actual evaluation and experience evaluation of geothermal resource advantage areas.

Compared with the weights of the two methods, the indicators with larger weights in PCA are Rifted basin, Metamorphic rock, Earth heat flow value, and Deep fault. The three indicators with larger weights in AHP are Silicon containing equation heat storage value, K/Mg cation temperature scale heat storage value, and Geothermal water temperature. The weight selection of the indicators is relatively different, and the focus of the indicators is quite different, which has obvious comparative significance. In this study, compared with the two methods, the indicator normalization weight of geothermal temperature, Earth heat flow value, and Rifted basin has a larger proportion, indicating that it has the most significant impact on the distribution of geothermal resources.

### Comprehensive evaluation

#### Comprehensive method

Comparing the analysis of the two results in Figs. 13 and 14, the evaluation results of PCA more clearly show the dominant geothermal area in the western border of Jiangxi Province, but AHP highlights the dominant geothermal area in the southeast of Jiangxi Province. However, the overall trend is similar, and the main distribution areas of the dominant areas are basically the same, which can be divided into 7 areas. On the whole, both evaluation methods clearly and intuitively reveal the distribution law of geothermal energy in Jiangxi. The geothermal indicator analysis can better reflect the geothermal development value and potential of these regions. Both evaluation results reveal the distribution of geothermal resources in Jiangxi, with little difference. The main areas are also consistent with each other, indicating that the two methods not only play a role in comparison, but also can achieve mutual verification to a certain extent.

In order to better reflect the advantageous area of geothermal resources, the two result maps are superimposed on each other again by means of GIS. Mainly, the two are divided into 5 grades from good to bad, and assigned as Eq. (8). Then through GIS overlay, where the unit block value is 10–2, according to the grading of 10–9, 8–7, 6, 5–4, 3–2, it is divided into: advantage area, superior area, general area, inferior area, inferior area, a total of 5 types of geothermal resource divisions (Table 6), the formula is as follows Eq. (8):

8 $$ ZH = \sum\limits_{i = 1}^{n} {\left( {AHP_{i} + PCA_{i} } \right)} $$

**Table 6:** Regional evaluation table for comprehensive evaluation.

Evaluation division	AHP evaluation result assignment	PCA evaluation result assignment	Comprehensive evaluation score
Advantage area	5	5	10–9
Superior area	4	4	8–7
General area	3	3	6
Inferior area	2	2	5–4
Disadvantaged area	1	1	3–

In the formula: ZH is the comprehensive evaluation score of the geothermal resource advantageous area; AHP_i_ is the value of the AHP evaluation result of the evaluation unit, PCA_i_ assigns the PCA evaluation result of the evaluation unit.

Among them, the advantageous area and the superior area are the potential areas for geothermal development. The region is rich in resources and has great development value, which is suitable for large-scale and systematic geothermal resource development. In other areas, the relative resources are relatively poor, and the development and construction of geothermal directions can be considered according to the actual situation, and large-scale geothermal exploration and development is not recommended.

#### Results

**Figure 15:**
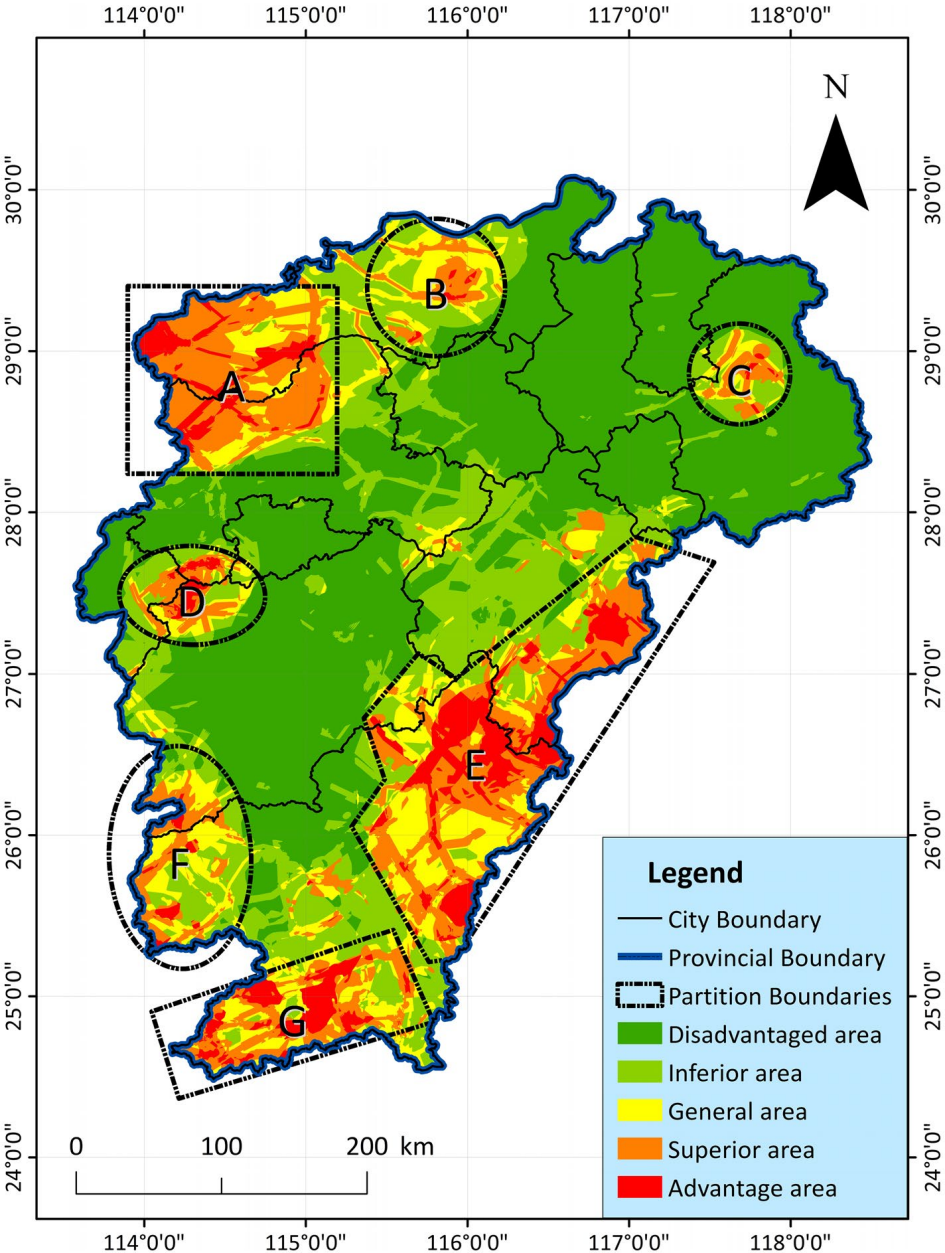
Distribution map for comprehensive evaluation of geothermal resource potential in Jiangxi Province, showing the final composite potential score for each cell.

By superimposing the evaluation results of the above two methods, it can be clearly seen that Jiangxi can be divided into 7 geothermal resource advantageous areas. It is mainly distributed in the southeastern border, western border and small northern areas of Jiangxi, and these areas have higher comprehensive evaluation scores. Judging from the evaluation results of various indicators and different methods, these 7 regions almost include the current geothermal resource advantageous areas in Jiangxi, basically indicating the scope suitable for the development and utilization of geothermal resources (Fig. 15, Supplementary Table S3).

#### Description

(A) Xiushui geothermal area: A number of major heat-controlling and earthquake-generating faults are distributed in the Xiushui Basin. Many geothermal springs have been exposed, and geothermal wells with a temperature of 83 ℃ and a water output of 1200 m^3^/day have been found. The burial depth of the Curie gradually decreases, and reaches a low value at the junction of Hubei and Jiangxi, indicating that the geothermal resource potential of this area gradually increases from southeast to northwest.
(B) Lushan geothermal area: Located in the Jiuling thermal uplift, the folded basement in the area is widely exposed, and the sedimentary caprock is extremely underdeveloped. There are developed faults in the area, and there are 3 important north–north-east conducting thermal fault zones. The magmatic activity is strong and frequent, and the magmatic activity in the Jinning and Yanshan periods is the most developed. There are 8 exposed hot springs in this area, all of which are related to faults. There are high stress zones and high anomalous geothermal gradients. Great potential and broad development prospects.
(C) Dexing geothermal area: Distributed in small-scale faulted basins, with only a few small active faults. The area belongs to the Dexing-Wuyuan dynamic metamorphic rock belt, the terrestrial heat flow value is above 70 mW/m^3^ on average, and the geothermal temperature is relatively high. Curie and Moho are all in this area indicating shallow depth and a few geothermal hot springs are found. The heat storage value of the silicon enthalpy equation is particularly high, which indicates that the geothermal resources are rich and high temperature geothermal exists.
(D) Wugongshan geothermal area: It is located in the thermal uplift of Wugong Mountain, with strong basement folds, relatively developed faults, and strong magmatic activity. The Caledonian rock base in Wugongshan is the largest in scale, and a marginal migmatite belt is developed. There are many hot springs exposed, with a maximum temperature of 77 ℃, which is of great development value.
(E) Huichang-Shicheng-Ningdu-Guangchang-Lichuan geothermal area: The geothermal area is located on the western slope of Wuyi Mountain, and the fault zone in the area controls many hot springs. And the magmatic rocks and thermal metamorphic rocks of many ages are widely distributed. The highest geothermal water of 83 °C has been detected, and the area has considerable potential for geothermal water resources. And the terrestrial heat flow value in the area belongs to the highest area in Jiangxi.
(F) Chongyi-Sichuan geothermal area: The regional calculated heat storage value is relatively high, and the average terrestrial heat flow value is above 70 mW/m^3^. There are magmatic rocks of multiple periods in the area, and there are many radioactive rock bodies. There are many cross-active faults in the area, and dozens of hot springs are found, all of which indicate that the geothermal resources here are rich, and the development value and potential are great.
(G) Xunwu-Dingnan-Longnan geothermal area: There are multiple fault zones and multiple hot springs nearby, surrounded by multiple volcanic basins. Moreover, there are volcanic rocks of multiple ages exposed, and it is the shallowest area in Jiangxi Province. There are geothermal anomalies in view of the geothermal gradient. From east to west, the radioactivity of the magmatic rocks also increases gradually, which has a good prospect for geothermal exploration.

#### High-value area of geothermal potential

**Figure 16:**
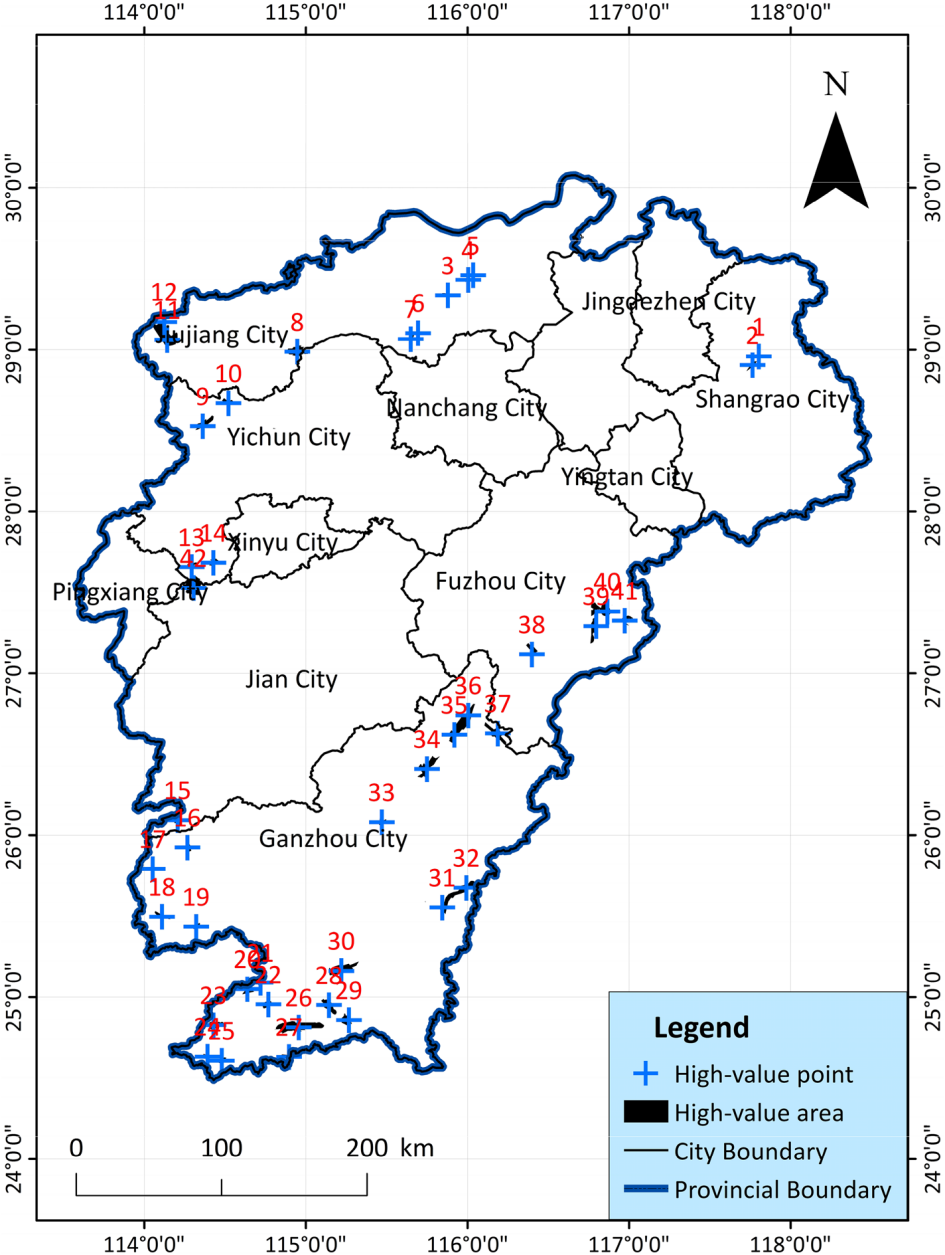
Distribution map of comprehensive evaluation of geothermal resource potential and distribution of high value points in Jiangxi Province, showing the percentage ranking for each indicator.

In order to more accurately find the areas with the most abundant geothermal resources in each region, the top 40 unit blocks with higher scores are determined as high-value areas of geothermal potential based on the results of the comprehensive potential evaluation. In order to find the corresponding coordinates more accurately, on the basis of the high value area, the coordinate point of the relative center is determined as the high value point(Fig. 16). And count the indicator data of each high value point, and organize it into detailed data Supplementary Tables S4 and S5.

According to the different conditions of the 7 major advantageous areas, 2 to 11 high-value areas are divided in each resource advantageous area, and the coordinate points are determined to facilitate more accurate development planning. On the basis of geothermal resource advantageous areas, typical high-value areas are further subdivided according to the comprehensive score value. The typical high-value areas belong to the blocks with the highest comprehensive evaluation scores in the corresponding advantageous areas. The purpose of dividing more specific high-value areas is to facilitate more accurate planning and development by the regional government (Fig. 16). A total of 38 high-value areas and 42 abnormal points have been identified in Jiangxi, and each point is the most abundant geothermal resource in this area.

According to the comprehensive evaluation results, the advantages of geothermal resources are divided into three levels: The first-level advantageous resource areas are the Xiushui geothermal area near Jiujiang City, and the Ningdu-Guangchang geothermal area, near abnormal points 8, 11, 35, and 36. The second-level advantageous resource areas are the Wugong Mountain Thermal Area, the Dingnan-Anyuan Geothermal Area, and the Lichuan Geothermal Area, near abnormal points 13, 28, 29, 39, and 40. The third-level advantageous resource areas are Chongyi-Sichuan geothermal area, Lushan geothermal area and Dexing geothermal area, near abnormal points 1, 3, 4, 18, and 19. The remaining abnormally high score points are used as secondary development geothermal resource areas. It is recommended to develop geothermal resources according to the predominant level of geothermal resources. Prioritize planning and development from high-value areas for comprehensive evaluation of geothermal resources.

#### High value point indicator analysis

**Figure 17:**
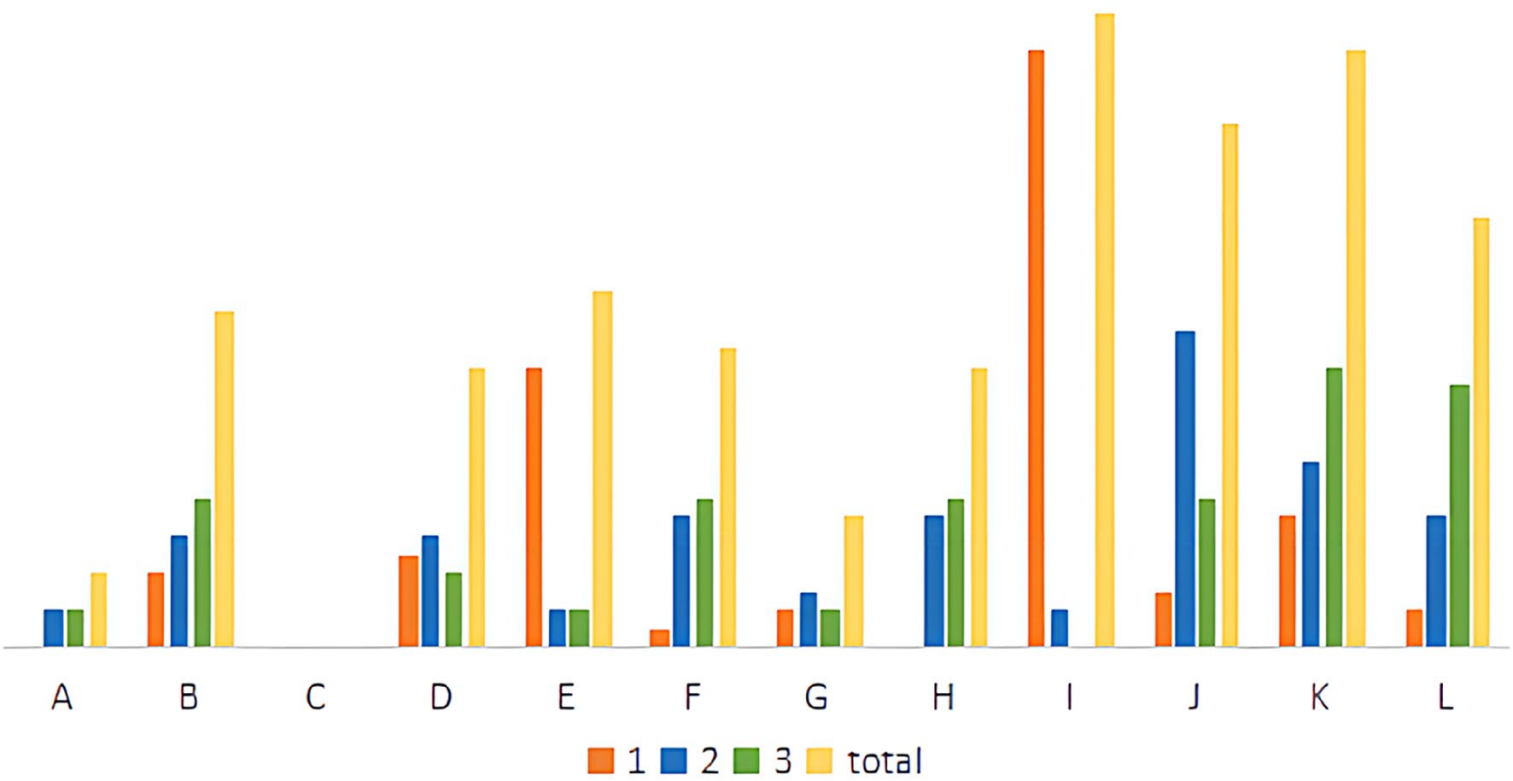
Comparison of the importance of geothermal high-value point indicators.

In order to analyze the main factors affecting the potential of geothermal resources, this article counts 42 geothermal hotspots with the highest value in each region. The indicator levels of each geothermal hotspot are divided into four levels: 1, 2, 3, and secondary, and the first three levels are selected as the important impact indicators of the geothermal high-value point, which are marked in yellow in Supplementary Table S5. Statistics of these 12 impact indicators in this sample data (Supplementary Tables S4 and S5), the sample data counts the location coordinates, evaluation scores and main influencing factors of 42 geo-hot spots. The percentage of importance of each indicator at each level is drawn as a chart as shown in Fig. 17 and Table 7.

**Table 7:** Statistical table of importance percentage of indicators.

Percent	1	2	3	total
A	0.00%	4.76%	4.76%	9.52%
B	9.52%	14.29%	19.05%	42.86%
C	0.00%	0.00%	0.00%	0.00%
D	11.90%	14.29%	9.52%	35.71%
E	35.71%	4.76%	4.76%	45.24%
F	2.38%	16.67%	19.05%	38.10%
G	4.76%	7.14%	4.76%	16.67%
H	0.00%	16.67%	19.05%	35.71%
I	76.19%	4.76%	0.00%	80.95%
J	7.14%	40.48%	19.05%	66.67%
K	16.67%	23.81%	35.71%	76.19%
L	4.76%	16.67%	33.33%	54.76%

The meanings of the codes in the Fig. 17 and Table 7 are as follows: A—Metamorphic rock, B—Earth heat flow value, C—Rifted basin, D—Radioactive heat generation of rocks, E—Active fault, F—Curie feature, G—Moho feature, H—Geothermal water temperature, I—Deep fault, J—Silicon containing equation heat storage value, K—Magmatic rock, L—K/Mg cation temperature scale heat storage value. In all data, divide according to important level, 1 indicates that the percentage of the first indicator; 2 indicates that the percentage of the second indicator; 3 indicates that the percentage of the third indicator; Total indicates that the comprehensive percentage of the top three.

According to the statistics of geothermal indicator information, it can accurately guide regional geothermal development and leave accurate data information for future geothermal research. Through the comparative analysis of the charts, it is known that the important influence indicators in the high-value area of geothermal evaluation are deep fault, silicon containing equation heat storage value and magmatic rock. And the indicator of deep fault has the highest percentage (76.19%, 80.95%) in the first-level importance. It is deduced from this that the primary indicator affecting the distribution of geothermal resources is deep fault. Based on the analysis of comprehensive geological data, it is easier to explore geothermal resources in areas surrounding deep faults.

**Figure 18:**
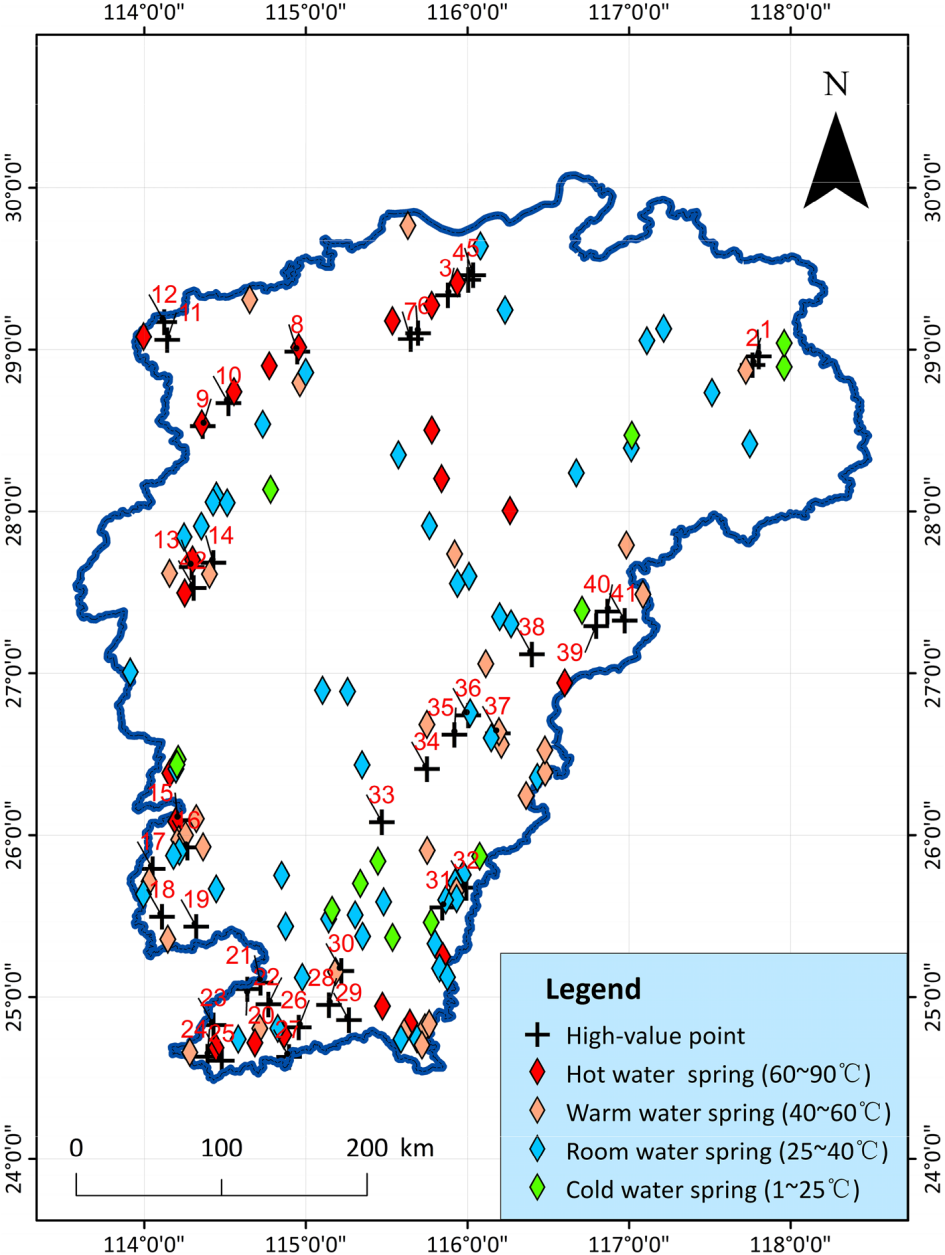
Verification and comparison of geothermal high-value points, showing the location of existing and presumed hotspots.

In order to compare the existing geothermal hot springs, boreholes and inferred geothermal high-value points, the superimposed results are especially verified in Fig. 18. The map includes the discovered hot spring sites and drilling sites, respectively. The water temperature is roughly divided into four categories: hot water, warm water, warm water, and cold water, as well as the presumed high-value hot spots. From the comparison in the figure, it can be seen that the geothermal predictions of the Wugong Mountain in the central area of the western border of Jiangxi, the Dingnan area in the south, and the Guangchang and Shicheng areas in the east are very well fitted, and are basically similar to the locations of the discovered geothermal springs. There are also many discovered geothermal springs near other high-value areas, and comparison and verification can provide a clearer understanding of the accuracy of this study.

## Discussion

GIS-based regional-scale geothermal potential mapping research has been applied in many countries^72–75^, but it has not been widely used in underdeveloped areas. This is not necessarily due to lack of resources, but due to lack of geospatial data. The spatial data analysis presented here not only provides the ability to delineate areas within an area where geothermal resources may exist, but also has the opportunity to improve our understanding of why geothermal resources do not exist everywhere. In the weight of evidence approach, geological evidence is combined in a multiplicative manner. Therefore, in terms of spatial evidence integration, the application is similar to that of fuzzy sets^16^ or weighted indices^76^.

Jiangxi Province has complete geological data, including geophysical exploration, hydrochemical analysis, hydrological drilling and so on. In addition, there are many geothermal springs in the study area, some of which are artificial borehole hot springs, and their quality and quantity are more suitable for regional and prospect-scale mapping. In the selection of indicators, the geological point of view, lithology, geological structure, and spatial distribution characteristics of magmatic rocks, etc. are analyzed; The data of temperature field, magnetic field and gravity field were obtained by means of geophysics. Among them, many factors can clearly reflect the distribution of geothermal: spatial distribution of geothermal water temperature, geothermal heat flow, Curie surface characteristics, Moho surface characteristics, deep fault inferred from gravity anomalies, etc.; There are also inferred geothermal water temperature, cation temperature scale, and thermal storage value of silicon enthalpy equation method to obtain accurate underground hot water temperature and other detailed information. This study has proved that an evaluation model can be established with the support of AHP and PCA analysis, and the results of the geothermal resource potential evaluation can be obtained by combining the results of the two, which can further clarify the development direction and scope of geothermal resources in the future. Jiangxi can be divided into 7 geothermal resource advantageous areas, mainly distributed in the southeastern border, western border and northern area of Jiangxi. The comprehensive evaluation scores of these areas are relatively high, which basically indicates the scope suitable for the exploration, development and utilization of geothermal resources.

In this study, we cross-validate the predicted high-potential area with the actual geothermal field, confirming the importance of the correctness of the potential evaluation. On the basis of this achievement, we also conducted an indicator analysis on the high-value areas of geothermal potential evaluation, and counted the geothermal hotspots with higher scores. According to the indicator assignment and grading in the evaluation unit, after horizontal comparison, the importance of each indicator is analyzed. Therefore, it is deduced that the heat storage value of deep fault, magmatic rocks and silicon enthalpy equations, among which deep fault account for the largest proportion. These three indicators are the indication indicators reflecting the distribution of geothermal resources.

The evaluation method of the GIS model can be used to predict the distribution of geothermal resources, and the actual effect has been verified. The weight selection method combined with AHP and PCA method has indeed improved the evaluation accuracy, and there is still a lot of room for research in this direction. However, the fineness is not enough, and accurate detection in a small area still needs to be supported by drilling data.

## Supplementary Information

Supplementary Table S1.

Supplementary Table S2.

Supplementary Table S3.

Supplementary Table S4.

Supplementary Table S5.

Supplementary Information 6.

## Data Availability

All data generated or analysed during this study are included in this published article [and its supplementary information files].
